# Weekly Training Frequency Effects on Strength Gain: A Meta-Analysis

**DOI:** 10.1186/s40798-018-0149-9

**Published:** 2018-08-03

**Authors:** Grant W. Ralston, Lon Kilgore, Frank B. Wyatt, Duncan Buchan, Julien S. Baker

**Affiliations:** 1 Institute for Clinical Exercise and Health Science, Applied Physiology Research Laboratory, School of Science and Sport, University of the West of Scotland, Hamilton, Lanarkshire ML3 0JB Scotland, UK; 2 Kilgore Academy, Azle, TX USA; 3 Department of Athletic Training and Exercise Physiology, Midwestern State University, Wichita Falls, TX USA

**Keywords:** Strength training frequency, Resistance training frequency for strength development

## Abstract

**Background:**

The current recommendations for resistance training (RT) frequency range from 2 to 5 days per week (days week^− 1^) depending on the subjects’ training status. However, the relationship between RT frequency and muscular strength remains controversial with reported variances existing across different population groups. We conducted a meta-analysis that (1) quantified the effects of low (LF; 1 day week^− 1^), medium (MF; 2 days week^− 1^), or high (HF; ≥ 3 days week^− 1^) RT frequency on muscular strength per exercise; (2) examined the effects of different RT frequency on one repetition maximum (1RM) strength gain profiles (multi-joint exercises and single joint exercises); (3) examined the effects of different RT frequency on 1RM strength gain when RT volume is equated; and (4) examined the effects of different RT frequency on 1RM strength gains on upper and lower body.

**Methods:**

Computerised searches were performed using the terms ‘strength training frequency’, ‘resistance training frequency’, ‘training frequency’, and ‘weekly training frequency’. After review, 12 studies were deemed suitable according to pre-set eligibility criteria. Primary data were pooled using a random-effects model. Outcomes analysed for main effects were pre- to post strength change with volume-equated studies that combined multi-joint and isolation exercise; isolation-only exercise and untrained subjects only. Heterogeneity between studies was assessed using *I*^2^ and Cochran’s Q statistics with funnel plots used to assess publication bias and sensitivity analyses calculated for subgroups.

**Results:**

Pre- versus post-training strength analysis comprised of 74 treatment groups from 12 studies. For combined multi-joint and isolation exercises, there was a trend towards higher RT frequency compared with lower frequency [mean effect size (ES) 0.09 (95% CI − 0.06–0.24)] however not significant (*p* = 0.25). Volume-equated pre- to post-intervention strength gain was similar when LF was compared to HF [mean ES 0.03 (95% CI − 0.20–0.27); *p* = 0.78]. Upper body pre- to post-intervention strength gain was greater when HF was compared with LF [mean ES 0.48 (95% CI 0.20–0.76)] with significant differences between frequencies (*p* < 0.01). Upper body pre- to post-intervention strength gain was similar when MF was compared with LF (ES 0.12; 95% CI − 0.22–0.47); *p* = 0.48]. There was no significant difference in lower body mean ES between HF and LF [mean ES 0.21(95% CI − 0.55–0.13); *p* = 0.22]. There was a trend towards a difference in mean ES between MF and HF [mean ES 0.41(95% CI − 0.26–1.09); however, the effect was not significant (*p* = 0.23).

**Conclusions:**

The existing data does not provide a strong correlation between increased weekly training frequency (HF) and maximal strength gain in upper and lower body resistance exercises for a mixed population group. When RT is volume-equated for combined multi-joint and isolation exercises, there is no significant effect of RT frequency on muscular strength gain. More investigations are required to explore the effects of varying weekly training frequencies adequately.

## Key points

- When resistance training (RT) are equated for weekly training volume, low frequency (LF; 1 days week^− 1^), and high frequency (HF; ≥ 3 days week^− 1^) produce similar strength gains in combined multi-joint strength and isolation exercises.
- The use of LF training may be an appropriate intersession frequency dose to produce strength gains for untrained or older individuals. However, for muscular strength progression, the use of HF training can be used as an effective method of increasing weekly training volume that may contribute to an increase in strength.
- These findings also suggest that due to the absence of quality experimental data, it remains unclear whether RT frequency on its own has effects on muscular strength. More investigations and replication studies using appropriate study designs and comparable subject samples are required to explore the effects of different weekly training frequencies.

## Background

Resistance training (RT) has been acknowledged as an effective method to improve muscular strength, power, and hypertrophy which are fundamental components of physical fitness related to the quality of life [1]. Research over the last few decades has investigated the effects of several acute training variables on maximal strength gains that influence the overall outcome of an RT program [2–4]. These RT variables include exercise order, the number of sets, repetitions, inter-set recovery periods, training intensity per muscle group, and total training volume. Steib et al. [5] remarked that a training variable that has received limited consideration is RT frequency. The RT frequency is conditional on other training variables and individual’s ability to physically adapt from the mechanical stress placed upon the body [6]. Kraemer and Ratamess [7] defined RT frequency as several sessions performed during a specific time frame. Considerations towards intersession recovery are needed, as individuals exposed to excessive and frequent RT stimuli to the same muscle or groups of muscles could lead to over-training and a decrease in strength [8]. Equally, subjects that have unnecessary intersession recovery may have a detrimental effect on muscular strength due to detraining [9].

Quantifying recovery rates and providing recommendations on RT frequency for strength gains is difficult and may vary between training status, sex, and muscle groups. Physical activity guidelines from leading organisations including the American College of Sports Medicine (ACSM) [10] recommend RT frequency of 2 to 3 days per week (days week^− 1^) for healthy adults. These frequency recommendations are however notional, derived from conjecture rather than robust scientific evidence. This lack of evidence weakens established recommendations regarding progressive RT loading and training volumes for improved muscular strength [11]. Several RT studies have reported that an RT frequency of 2 to 3 days week^− 1^ for previously untrained individuals’ [12–15] produces optimum strength gains. Feigenbaum and Pollock [13] suggest that a single set program of 15 repetitions performed at a frequency of 2 to 3 days week^− 1^ allows for sufficient regeneration, while still providing 80–90% strength gains of more frequent RT programs. Moreover, the authors suggest that each RT session should be comprised of 8 to 10 different exercises that target the major muscles. However, these recommendations are generalised and are provided for all subjects with varying health conditions and age ranges.

RT studies frequently cited in scientific literature do not adequately describe the frequency for different population groups (untrained, trained, and well trained). For example, an often-cited study by McKenzie Gillam et al. [16] examined strength gains on untrained males when performing bench press lifts at either 1, 2, 3, 4, or 5 days week^− 1^ for 8 weeks. The results suggested that training 5 days week^− 1^ had greater strength improvements than the other groups (1, 2, 3, and 4 days week^− 1^, respectively). However, the total weekly volume between groups was not equalised with higher weekly frequency groups having significantly increased training volume at the end of the 9-week period. These findings are in contrast with McLester et al. [17] who investigated the effects of whole body RT programs on the strength of experienced weight trainers. Subjects trained either 1 or 3 days week^− 1^ with no significant differences between groups on eight out of nine strength measures, suggesting that 1 day week^− 1^ may be as effective as 3 days week^− 1^.

Unfortunately, there is limited meta-analytical evidence available due to the lack of available studies. However, a meta-analysis by Silva et al. [18] on adults over 55 years of age found no differences in strength when training 1, 2, or 3 days week^− 1^. The authors suggest that different combinations of acute RT variables might be equally valid in the strength development of healthy sedentary older adults. The only training variable that displayed any significant effect size (ES) for strength was training duration. A recent meta-analysis by Grgic et al. [19] compared different RT weekly frequencies (1, 2, 3, and ≥ 4 days week^− 1^) on muscular strength gains. The results of their analysis indicated a significant effect on muscular strength when weekly RT frequency was increased. However, when subgroup analysis was performed on volume-equated studies, no significant effect (*p* = 0.421) of RT training frequency on muscular strength gains was observed. Grgic et al. [19] concluded that higher frequency could be used as a method of increasing total weekly training volume to promote muscular strength gains.

The strength of evidence is currently restricted and as such has created academic debate on what constitutes the most effective weekly RT frequency for increasing muscular strength. Limited meta-analyses have been published that examine the effects of weekly training frequency on strength gains. Therefore, the purpose of this paper was to conduct a meta-analysis that (1) quantified the effects of low (LF; 1 day week^− 1^), medium (MF; 2 days week^− 1^), or high (HF; ≥ 3 days week^− 1^) RT frequency on muscular strength per exercise; (2) examined the effects of different RT frequency on one repetition maximum (1RM) strength gain profiles (multi-joint exercises and single joint exercises); (3) examined the effects of different RT frequency on 1RM strength gain when RT volume is equated; and (4) examined the effects of different RT frequency on 1RM strength gains on upper and lower body. Based on evidence generated from recent studies on RT frequency [20–23] and meta-analytic data [19], we hypothesised that no significant muscular strength difference exists between lower and higher RT frequency.

## Methods

### Literature Search

A systematic search of the literature was conducted by the recommendations and criteria outlined in the Preferred Reporting Items for Systematic Reviews and Meta-Analyses (PRISMA) statement [24]. Computerised searches were performed and generated citation lists from the following databases: PubMed, MEDLINE, SWETSWISE, EMBASE, SPORTDiscus™. The period of search history examined was inclusive to March 2018. Other relevant studies were identified by hand searching and cross-referencing of journals, reference lists, and other sources. Applicable descriptive terms that were used to retrieve studies in English included ‘strength training frequency’, ‘resistance training frequency’, ‘training frequency’, and ‘weekly training frequency’. Boolean operators, including AND, OR and NOT, were used to focus literature searches. The literature searches were limited to RT studies involving humans only. As a result, papers were retrieved from 1985 through to March 2018 in which one versus multiple days week^1^ RT frequencies were compared, in both untrained and trained male and female subjects. After performing the initial literature search, reference lists of articles retrieved were screened for any additional articles of relevance to the topic. Citations and abstracts from scientific conferences and studies published in foreign language journals were excluded.

### Eligibility Criteria

For inclusion in the meta-analysis, published experimental reports were required to present the following criteria: (a) RT program lasting a minimum duration of 4 weeks; (b) training at least one primary muscle group—pectoralis major, latissimus dorsi; deltoids (anterior, lateral, posterior); hamstrings (bicep femoris, semitendinosus, semimembranosus); biceps, or triceps; quadriceps (vastus medialis, vastus intermedius, vastus lateralis, rectus femoris); (c) adult male or female subjects between 18 and 75 years; (d) direct comparison of different weekly RT frequencies in traditional dynamic exercise using coupled concentric and eccentric actions; (e) subjects free from muscular-skeletal, or orthopaedic injuries, or physical limitations; (f) at least one measure of muscular strength conducted pre- to post; (g) subject’s descriptive characteristics included in report (height, weight, training status, and training experience); and (h) sufficient data to determine RT frequency to calculate ES. This analysis included randomised trials (RAN) and randomised control trials (RCTs) that observed the intervention treatments using stratified LF versus either MF or HF RT frequency. RAN allocation ensures no systematic differences between the intervention groups; however, no control group may impact upon the assessment of outcomes. RCTs are a more rigorous method for determining a cause-effect relationship between treatment and outcome.

### Search Strategy

Three reviewers (GR, LK, and DB) independently evaluated titles and abstracts of retrieved articles. Abstracts that did not provide sufficient information concerning the inclusion and exclusion criteria were retrieved for full-text evaluation. Each of the three reviewers independently evaluated full-text articles and determined the eligibility for this analysis. Each investigator individually conducted data extraction from eligible studies. If primary data was not available, then attempts were made to communicate with all authors. Unfortunately, no correspondence was made about obtaining primary data from the authors. This, therefore, resulted in data extraction using WebPlot-Digitizer (Web Plot Digitizer, V.3.11. TX, USA: Ankit Rohatgi, 2017). Where differences between the three reviewers (GR, LK, and DB) existed then further discussions and agreements were made by consensus. Post hoc reassessment of eight randomly selected studies was performed and the extracted results compared. Coder drift was < 10% in all cases for each investigator, and inter-rater (GR, LK, and DB) reliability was > 95%. The main categories of variables encoded were (1) descriptive characteristics of subjects, including age, RT experience, and sample size; (2) RT programme characteristics, including weekly training frequency, total training duration, number of sets per exercise, and number of reps per exercise; (3) measurement of strength outcome(s); and (4) treatment effects (mean and SD values of changes in strength outcomes for baseline and post-intervention in training and control groups).

### Assessment of Methodological Quality of Studies

Studies were rated using the Physiotherapy Evidence Database (PEDro) scale [25, 26] (Table 2). The scale has 11 criteria, with a maximum score of 10 for the PEDro scale. However, considerations were made, as the therapists, assessors, and technicians delivering interventions cannot be blinded; therefore, the maximum score for the PEDro scale, in this case, is nine. Studies with PEDro scores of ≥ 4 were considered as having adequate internal validity and, were included in the analysis. Three reviewers (GR, LK, and DB) independently assessed methodologic quality. Differences of opinion regarding the scoring of articles were resolved between the three investigators through discussion and consensus.

A meta-analysis was performed, whereby descriptive statistics were calculated to summarise and explain the results of the systematic review process. To compare findings of each study individual characteristics and data were tabulated onto a spreadsheet (Microsoft, Redmond, WA, USA) for coding, management, review, and data reference for statistic entry. Descriptive statistics including sample size (*n*), mean (M), and standard deviations (SD) were taken from each study, to provide information for the mean differences in pre- to post-intervention between groups (e.g. LF, MF, and HF) on various strength outcomes. Muscular strength was considered a continuous data variable; therefore, the standardised mean difference (SMD) with 95% confidence intervals (CI) were used to determine ES measures (Table 3).

A SD score was calculated for each outcome variable by using Cohen’s *d* index of an individual ES (di = [M1–M2]/SDpi) [27], where *d* = effect size, *i* = individual study, M1 = pre-intervention mean, M2 = post-intervention mean, and SDp = pooled standard deviation. The SD was calculated by summing the reported pre-intervention and post-intervention SDs and dividing by two. When the standard error of measurement (SEM) of the mean was specified, the SD was calculated according to the formula (SD = SEM*square root of N) [28]. Individual ES were weighted to account for individual sample sizes. Where a study reported, exact *p* values for change in strength the SD of change was computed. For studies that did not report exact *p* values, the SD of change was calculated using the pre-and post-intervention SDs. A random-effects inverse variance (IV) using the DerSimonian-Laird method [29] was used with the effects measure of SMD due to studies being performed with varied populations and methods. If a study had various time-points, only the pre- to post intervention strength outcomes were retrieved and included in the analysis. These figures were then used to calculate ES estimates and confidence intervals. For each strength measure, an ES was calculated as the pre- to post intervention change, divided by the pre-intervention SD [30].

### Meta-Analyses

Meta-analyses were primarily performed using Meta-Essentials [31] with each row represented a specific ES for a treatment group. If there were multiple ES for a treatment group, then each ES was coded in a separate row. This allowed for calculations of ES, SEM, and study size to assign appropriate weight to each study, and estimate a study effect. The final analysis was conducted using Review Manager (RevMan) version 5.3.5 for all other statistical analyses and forest plots. The difference in SD of post-intervention strength outcomes was computed using RevMan (version 5.3.5). Data required were either (1) means and SDs (pre-and post-intervention); (2) CI data for pre- to post-intervention change for each group or when this was unavailable; (3) *p* values for pre- to post-intervention change for each treatment group or if only the level of significance was available and, (4) default *p* values (e.g. *p* ≤ 0.05 becomes *p* ≤ 0.49, *p* ≤ 0.01 becomes *p* ≤ 0.0099 and *p* ≤ not significant becomes *p* ≤ 0.05).

### Heterogeneity and Risk of Bias

Cochran Q statistic [32] and *I*^2^ index tests were used to assess heterogeneity between studies. The Cochran Q statistic (Q) is an appropriate test for larger meta-analyses and uses the sum of squared deviations of each estimate derived from the pooled estimate and weights the contribution of each study. The *p* values were achieved by comparing the Q statistic with an *X*^2^ distribution with *k*^− 1^ degrees of freedom, where *k* represents the number of included studies. The *I*^2^ statistic was also used to assess heterogeneity, with an *I*^2^ > 50% applied to indicate heterogeneity. Treatment effects for muscular strength were calculated for each included study following coding of pre- to post changes and standard deviations (SDs). The ES of ≤ 0.2, ≤ 0.5, ≤ 0.8, and ≥ 0.8 were considered trivial, small, moderate, and large, respectively [26]. The degree of heterogeneity was assessed with the *I*^2^ test for each outcome. Non-significance indicates that the results of the different studies were similar (*p* ≥ 0.05).

Publication bias was evaluated by combining a funnel plot assessment with Duval and Tweedie’s [33] trim and fill correction. Trim and fill funnel plots were performed to assess for publication bias of literature in all comparison models. This was to ensure that included studies did not report an inflated account of the effect on training frequency and strength outcome. Forest plots were generated to show the study-specific ES and the respective CI. Each forest plots performance measure was visually inspected against its SE to account for the ‘file drawer problem’. This is the potential effect of published studies being intrinsically biased due to a higher probability of significant results.

Separate meta-regressions on ES were performed with the following moderators, including (1) multi-joint or single-joint exercise on 1RM strength gains; (2) volume-equated RT; and (3) upper and lower body strength gains. If insufficient data was available, then training frequencies were classified as either lower or higher RT frequency. When a study had three comparison groups (LF, MF, or HF), the highest frequency groups (MF and HF) were combined and classified as ‘higher’, and the lower frequency (LF) group classified as ‘lower’. In the regression model, mean differences in ES were calculated for each study to yield a study-level ES for the difference between LF, MF, and HF allowing for the generation of forest plots. Sensitivity analysis was performed, identifying any highly influential studies which might bias the analysis. This was performed for each model by removing one study at a time and then examining the weekly frequency volume predictor. Influential studies were identified and removed if it resulted in a change from significant (*p* ≤ 0.10) to nonsignificant (*p* ≥ 0.10), or vice versa, or if removal caused a substantial change in the magnitude of the coefficient.

## Results

**Fig. 1:**
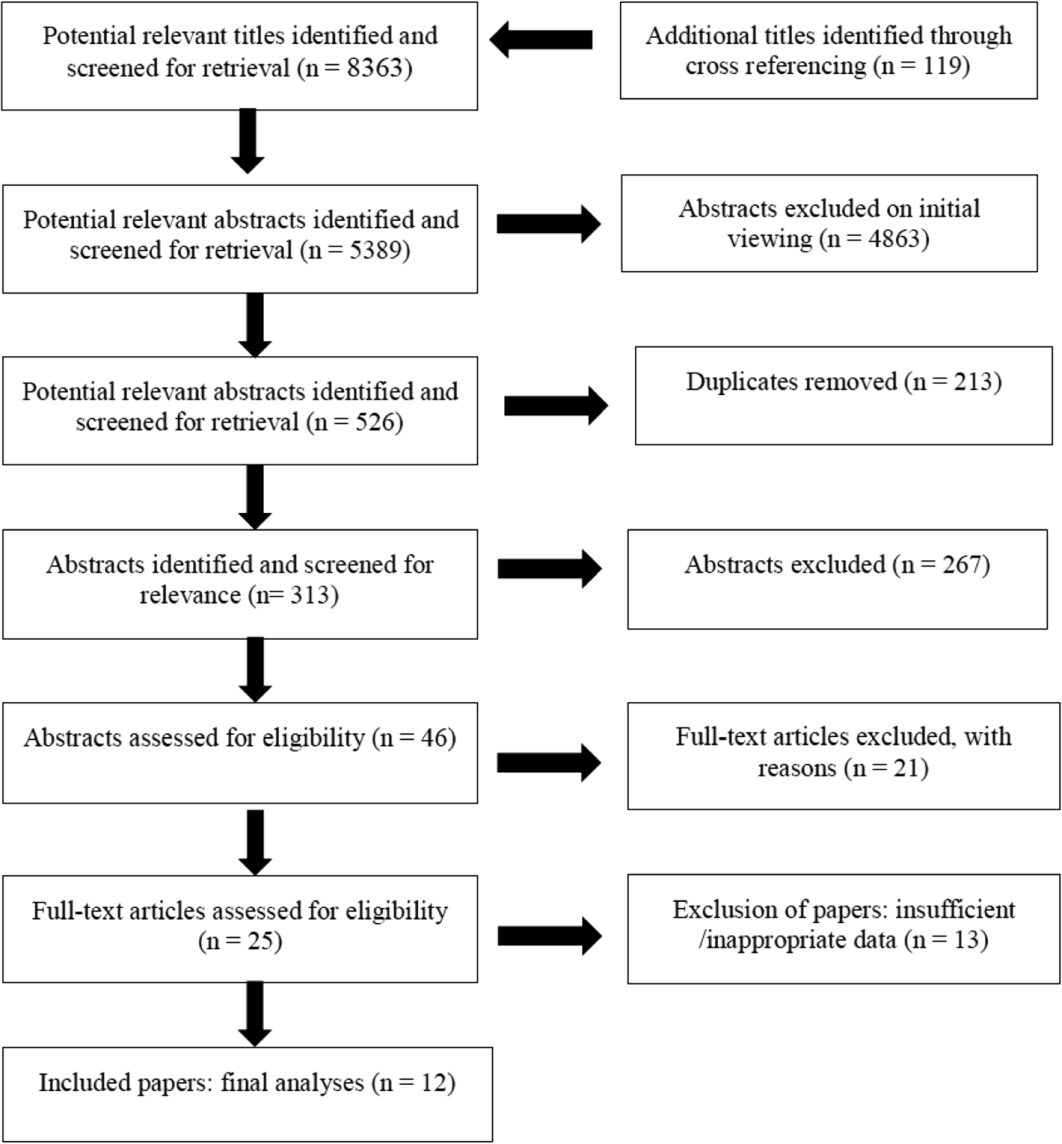
The flow of journal articles through the systematic review process

The flow of literature search and selection is depicted in Fig. 1 from ‘potentially relevant’ to final article inclusion.

### Study Selection

The preliminary search yielded 8363 relevant abstracts and citations. Twenty-five potential papers from the primary analysis were screened for content relevance. Following the inclusion and exclusion criteria (Table 1), 6 of the 25 publications on weekly RT frequency were excluded [34–39] in the analysis. Descriptions for the exclusion of six of the 25 studies (Table 2) included; publications assessing the same weekly HF only [36–39]; or omitted if exercises primarily engaged the cervical and lumbar muscles [34, 35], as these muscles relate to both upper and lower body limbs, presenting a confounding influence.

**Table 1:** Inclusion and exclusion criteria

Inclusion criteria	Exclusion criteria
Strength assessment of one or more muscle groups used (isolation exercises, e.g. leg extension with stress gauge).	Small subject sample groups (e.g. *n* < 6)
Minimum duration of training intervention is 3 weeks; longitudinal studies would be preferred (greater than 12 weeks).	Legal or illegal ergogenic aids or supplementation has been used during interventions.
Preferred if control group included within research design with subjects randomly assigned to groups.	Variation within the training order throughout the weeks.
RT program supervised with the RT intervention of similar order and if applicable inter-set recovery periods standardised for multiple sets.	No quasi RCT or narrative studies/reviews to be included.
Conducted warm-up is standardised between groups.	Subjects below 18 years of age.
Subjects trained to volitional fatigue with appropriate criteria regarding training intensity.	Did not report results adequately (pre- to post-mean and standard deviation).
Comparison of one vs. two, and ≥ three training session per week.	Examined the effects of concurrent training (i.e. combined RT and endurance training).
	Investigated the effects of nutritional supplements in combination with RT.
	Concurrent aerobic and strength training interventions.

**Table 2:** Methodological quality of studies based on the PEDro score

Author (ref)	PEDro scale item											Total
	1^a^	2	3	4	5	6	7	8	9	10	11	
Carroll et al. [11]	Yes	1	0	1	0	0	0	1	1	1	1	6
McLester et al. [17]	Yes	1	0	1	0	0	0	1	1	1	1	6
Brigatto et al. [22]	Yes	1	0	1	0	0	0	1	1	1	1	6
Gentil et al. [44]	Yes	1	0	1	0	0	0	1	1	1	1	6
Murlasits et al. [45]	Yes	1	0	1	0	0	0	1	1	1	1	6
Silva et al. [46]	Yes	0	0	1	0	0	0	1	1	1	1	5
DiFranciso-Donoghue et al. [47]	Yes	1	0	1	0	0	0	1	1	1	1	6
Lera Orsatti et al. [48]	Yes	0	0	1	0	0	0	0	1	1	1	4
Candow and Burke [49]	Yes	1	0	1	0	0	0	1	1	1	1	6
Schoenfeld et al. [50]	Yes	1	0	0	0	0	0	1	1	1	1	5
Thomas and Burns [51]	Yes	0	0	1	0	0	0	1	1	1	1	5
Arazi and Asadi [52]	Yes	1	0	0	0	0	0	1	1	1	1	5

*PEDro* Physiotherapy Evidence Database. The PEDro scale is based on the Delphi list [25]. Column 1^a^ not used in the calculation of the scores. Only criterion 2–11 are scored giving a total out of 10. Column numbers correspond to the following criteria on the PEDro scale: *1*^*a*^ = eligibility criteria (*1*^a^ = eligibility criteria specified [yes/no]); *2* = random allocation*; 3* = concealed allocation; 4 = groups similar at baseline; *5* = blinded subjects; *6* = blinded therapists; *7* = blinded assessors; *8* = follow-up measures obtained for > 85% of subjects; *9* = intention to treat analysis; *10* = between-groups statistical comparison; *11* = point measures and measures of variability

**Table 3:** Excluded studies from meta-analysis due to confounding factors

Study	*N*	Age, years (mean ± SD)	Duration	Testing modality	Sets (reps)	Days trained per week	Frequency per week	Pre- vs. post-intervention kg, mean ± SD	Pre- vs. post-intervention strength difference kg (% change)	*p* value (pre vs. post)	ES
Gomes et al. [20]	12 11	25.5 (± 1.25) 27.1 (± 1.85)	8 weeks	Sq	5–10 (8–12)	1 5	LF HF	132.9 ± 28.0 vs. 140.9 ± 25.5 123.3 ± 17.5 vs. 135.3 ± 22.2	8 (6.02) 12 (9.73)	0.47 0.17	0.30 0.60
Gomes et al. [20]	12 11	25.5 (± 1.25) 27.1 (± 1.85)	8 weeks	BP	5–10 (8–12)	1 5	LF HF	103.5 ± 15.4 vs. 109.1 ± 18.5 100.6 ± 14.5 vs. 110.3 ± 12.1	5.6 (5.41) 9.7 (9.64)	0.43 0.10	0.33 0.73
Serra et al. [21]	26 32 16	35.3 (± 1.75) 34 (± 2) 34.85 (± 0.85)	3 months	WG PullD	3 (10–20)	2 3 4	MF HF HF	42.6 ± 20.7 vs. 48.5 ± 20.7 44.4 ± 13.9 vs. 51.3 ± 13.9 40.6 ± 20.3 vs. 48.6 ± 21.8	5.9 (13.8) 6.9 (13.5) 8 (16.5)	≤ 0.001 ^a^ ≤ 0.001^a^ ≤ 0.001^a^	0.29 0.50 0.38
Serra et al. [21]	26 32 16	35.3 (± 1.75) 34 (± 2) 34.85 (± 0.85)	3 months	CP	3 (10–20)	2 3 4	MF HF^e^ HF	41.2 ± 25.6 vs. 49.9 ± 30.2 46.6 ± 20.8 vs. 59.7 ± 25.1 42.1 ± 27.9 vs. 55.6 ± 36.2	8.7 (21.1) 13.1 (28.1) 13.5 (32.1)	≤ 0.001^a^ ≤ 0.001^a^ ≤ 0.00^a^	0.31 0.57 0.42
Serra et al. [21]	26 32 16	35.3 (± 1.75) 34 (± 2) 34.85 (± 0.85)	3 months	LP	3 (10–20)	2 3 4	MF HF^e^ HF	108.9 ± 49.1 vs. 128.9 ± 54.5 120.2 ± 48.0 vs. 149.0 ± 57.7 128.8 ± 55.4 vs. 168.1 ± 89.0	20 (18.4) 28.8 (24.0) 39.3 (30.5)	≤ 0.001^a^ ≤ 0.001^a^ ≤ 0.001^a^	0.39 0.54 0.53
Yue et al. [23]	9 9	28 (± 7.9) 21 (± 3.2)	6 weeks	Sq	4 (8–12) 2 (8–12)	2 4	MF HF	103 ± 27 vs. 115 ± 34 90 ± 9 vs. 103 ± 13	12 (11.7) 13 (14.4)	≤ 0.001^a^ ≤ 0.001^a^	0.39 1.16
Yue et al. [23]	9 9	28 (± 7.9) 21 (± 3.2)	6 weeks	BP	4 (8–12) 2 (8–12)	2 4	MF HF	77 ± 27 vs. 88 ± 30 70 ± 17 vs. 81 ± 15	11 (14.3) 11 (15.7)	≤ 0.001^a^ ≤ 0.001^a^	0.39 0.69
Carpenter et al. [34]	10 12 12 7	31(± 31)	20 weeks	LumExt	1 (8–12)	0.5 2 3 4	LF MF HF HF	73.6 ± 24.7 vs. 98.2 ± 32.8 58.9 ± 18.0 vs. 86.2 ± 28.0 69.9 ± 27.5 vs. 105.9 ± 40.8 78.2 ± 21.4 vs. 115.8 ± 31.3	24.6 (33.4) 27.3 (46.3) 36 (51.5) 37.6 (48.1)	≤ 0.05^b^ ≤ 0.05^b^ ≤ 0.05^b^ ≤ 0.05^b^	0.85 1.16 1.03 1.40
Pollock et al. [35]	14 19	26 (± 9) 30 (± 9)	12 weeks	CervExt	1 (8–12)	1 2	LF MF	28.3 ± 6.7 vs. 38.2 ± 7.8 27.4 ± 8.5 vs. 38.2 ± 10.9	9.9 (35.0) 10.8 (40.9)	0.001^a^ 0.002^b^	1.36 1.10
Taaffe et al. [40]	11 12 11	68.5 (± 3.6) 69.4 (± 3.0) 71.0 (± 4.1)	24 weeks	LPull	3 (8)	1 2 3	LF MF HF	36.0 ± 14.2 vs. 45.3 ± 4.31 35.8 ± 9.7 vs. 47.0 ± 4.20 33.6 ± 10.9 vs. 48.9 ± 4.31	9.3 (25.83) 11.2 (31.28) 15.3 (45.54)	≤ 0.001^a^ ≤ 0.001^a^ ≤ 0.001^a^	0.89 1.50 1.85
Taaffe et al. [40]	11 12 11	68.5 (± 3.6) 69.4 (± 3.0) 71.0 (± 4.1)	24 weeks	BC	3 (8)	1 2 3	LF MF HF	16.5 ± 7.1 vs. 21.9 ± 2.99 17.0 ± 4.3 vs. 24.4 ± 2.77 16.7 ± 6.2 vs. 23.2 ± 2.99	5.4 (32.73) 7.4 (43.53) 6.5 (38.92)	≤ 0.001^a^ ≤ 0.001^a^ ≤ 0.001^a^	0.99 2.05 1.34
Taaffe et al. [40]	11 12 11	68.5 (± 3.6) 69.4 (± 3.0) 71.0 (± 4.1)	24 weeks	BExt	3 (8)	1 2 3	LF MF HF	62.3 ± 24.5 vs. 83.1 ± 9.17 63.4 ± 11.3 vs. 84.2 ± 9.70 65.0 ± 26.5 vs. 84.4 ± 9.29	20.8 (33.39) 20.8 (32.81) 19.4 (29.85)	≤ 0.001^a^ ≤ 0.001^a^ ≤ 0.001^a^	1.12 1.98 0.98
Taaffe et al. [40]	11 12 11	68.5 (± 3.6) 69.4 (± 3.0) 71.0 (± 4.1)	24 weeks	LP	3 (8)	1 2 3	LF MF HF	109.5 ± 29.8 vs. 135.2 ± 12.27 113.3 ± 19.3 vs. 142.8 ± 12.47 110.5 ± 29.5 vs. 145.1 ± 3.7	25.7 (23.47) 29.5 (26.04) 34.6 (31.31)	≤ 0.001^a^ ≤ 0.001^a^ ≤ 0.001^a^	1.13 1.82 1.65
Taaffe et al. [40]	11 12 11	68.5 (± 3.6) 69.4 (± 3.0) 71.0 (± 4.1)	24 weeks	KExt	3 (8)	1 2 3	LF MF HF	39.3 ± 15.4 vs. 67.1 ± 10.95 43.2 ± 8.6 vs. 72.6 ± 10.74 42.4 ± 12.4 vs. 62.6 ± 10.61	27.8 (70.74) 29.4 (68.06) 20.2 (47.64)	≤ 0.001^a^ ≤ 0.001^a^ ≤ 0.001^a^	2.08 3.02 1.75
Taaffe et al. [40]	10 12 11	68.5 (± 3.6) 69.4 (± 3.0) 71.0 (± 4.1)	24 weeks	KFlex	3 (8)	1 2 3	LF MF HF	16.3 ± 7.5 vs. 28.4 ± 4.43 19.6 ± 6.8 vs. 30.4 ± 4.50 20.0 ± 9.4 vs. 28.5 ± 4.64	12.1 (74.23) 10.8 (55.10) 8.5 (42.50)	≤ 0.001^a^ ≤ 0.001^a^ ≤ 0.001^a^	1.96 1.87 1.15
Farinatti et al. [41]	10 11 10	72 (± 5.0) 66 (± 7.0) 68 (± 4.0)	16 weeks	DumBP	1 (8–12)	1 2 3	LF MF HF	8.2 ± 1.8 vs. 13.0 ± 2.0 9.6 ± 2.2 vs. 14.4 ± 5.2 10.4 ± 0.9 vs. 15.6 ± 1.7	4.8 (58.54) 4.8 (50.0) 5.2 (50.0)	≤ 0.01^a^ ≤ 0.01^a^ ≤ 0.01^a^	2.52 1.20 3.82
Farinatti et al. [41]	10 11 10	72 (± 5.0) 66 (± 7.0) 68 (± 4.0)	16 weeks	DCur	1 (8–12)	1 2 3	LF MF HF	7.8 ± 1.5 vs. 12.2 ± 2.3 8.4 ± 1.7 vs. 13.6 ± 2.6 9.2 ± 1.8 vs. 15.6 ± 0.9	4.4 (56.41) 5.2 (61.90) 6.4 (69.57)	≤ 0.01^a^ ≤ 0.01^a^ ≤ 0.01^a^	2.27 2.37 4.50
Farinatti et al. [41]	10 11 10	72 (± 5.0) 66 (± 7.0) 68 (± 4.0)	16 weeks	KExt	1 (8–12)	1 2 3	LF MF HF	18.0 ± 3.7 vs. 25.6 ± 3.2 19.2 ± 4.6 ± 29.6 ± 7.4 19.8 ± 4.9 vs. 32.0 ± 6.8	7.6 (42.22) 10.4 (54.17) 12.2 (61.62)	≤ 0.01^a^ ≤ 0.01^a^ ≤ 0.01^a^	2.20 1.69 2.06
Farinatti et al. [42]	10 11 10	72 (± 5.0) 66 (± 7.0) 68 (± 4.0)	16 weeks	StCR	1 (8–12)	1 2 3	LF MF HF	12.0 ± 2.0 vs. 16.8 ± 1.9 12.2 ± 3.0 vs. 17.0 ± 3.7 11.8 ± 0.5 vs. 16.8 ± 1.5	4.8 (40.0) 4.8 (39.34) 5.0 (42.37)	≤ 0.01^a^ ≤ 0.01^a^ ≤ 0.01^a^	2.46 1.43 4.47
Burt et al. [42]	10 11	Students	8 weeks.	LP	1 (6–10)	1 2	LF MF	263.31 ± 37.23 vs. 362.65 ± 51.06 244.89 ± 45.80 vs. 391.53 ± 45.18	99.34 (99.34) 146.64 (59.9)	≤ 0.0001^a^ ≤ 0.0001^a^	2.22 3.22
Serra et al. [67]	18 17 10	41.4 (± 9.9)	4 months 8 months	BP	3 (10–12)	2 3 4	MF HF HF	41.7 ± 16.4 vs. 57.4 ± 15.4 38.8 ± 16.3 vs. 59.5 ± 16.5 39.0 ± 16.1 vs. 62.8 ± 18.1	36.3 (37.6) 46.5 (53.4) 65.1 (61.0)	0.01^a^ 0.001^a^ 0.01^a^	0.99 1.26 1.39
Serra et al. [67]	18 17 10	41.4 (± 9.9)	4 months 8 months	LP	3 (10–12)	2 3 4	MF HF HF	83.3 ± 21.4 vs. 113.3 ± 26.6 88.2 ± 21.9 vs. 112.5 ± 18.5 80.0 ± 25.4 vs. 117.8 ± 30.5	30.4 (36.01) 32.4 (27.6) 45.4 (47.3)	0.001^a^ ≤ 0.001^a^ 0.01^a^	1.24 1.20 1.35
Serra et al. [67]	18 17 10	41.4 (± 9.9)	4 months 8 months	LPull	3 (10–12)	2 3 4	MF HF HF	50.1 ± 12.0 vs. 67.4 ± 12.0 52.2 ± 12.8 vs. 69.7 ± 17.0 54.5 ± 16.2 vs. 77.5 ± 18.1	29.4 (34.5) 32.0 (33.5) 46.0 (42.2)	0.0001^a^ 0.002^a^ 0.01^a^	1.44 1.16 1.34
Benton et al. [36]	11 10	47.6 (± 1.2)	8 weeks	CP	3–6 (8–12)	3 4	HF HF	37.4 ± 2.5 vs. 50.0 ± 3.5 44.5 ± 3.0 vs. 59.7 ± 4.1	12.6 (34.0) 15.2 (34.2)	≤ 0.001^a^ ≤ 0.001^a^	1.25 1.34
Benton et al. [36]	11 10	47.6 (± 1.2)	8 weeks	LP	3–6 (8–12)	3 4	HF HF	105.1 ± 9.8 vs. 135.7 ± 16.7 119.7 ± 11.5 vs. 178.6 ± 19.6	30.6 (29.1) 58.9 (49.2)	≤ 0.001^a^ ≤ 0.001^a^	0.67 1.16
Ribeiro et al. [37]	5 5	26.7 (± 2.7)	4 weeks	BP	4 (6–20)	4 6	HF HF	104.4 ± 19.3 vs. 113.2 ± 23.0 105.6 ± 16.4 vs. 117.6 ± 16.3	8.8 (8.4) 12 (11.4)	0.53 0.28	0.41 0.73
Hoffman et al. [38]	12 15 23 11	19.7 ± (1.4) 20.1 ± (1.5) 20.1 ± (1.1) 19.7 ± (1.1)	10 weeks.	BP	1–5 (2–10)	3 4 5 6	HF HF HF HF	107.2 ± 11.6 vs. 109.1 ± 28.7 127.7 ± 13.9 vs. 132.2 ± 14.5 131.1 ± 20.1 vs. 135.3 ± 19.0 143.9 ± 12.0 vs. 149.7 ± 17.3	1.9 (1.8) 4.5 (3.5) 4.2 (3.2) 5.8 (4.0)	0.83 0.39 0.47 0.37	0.09 0.32 0.21 0.39
Hoffman et al. [38]	12 15 23 11	19.7 ± (1.4) 20.1 ± (1.5) 20.1 ± (1.1) 19.7 ± (1.1)	10 weeks.	Sq	1–5 (2–10)	3 4 5 6	HF HF HF HF	140.1 ± 18.6 vs. 147.4 ± 38.9 173.6 ± 36.2 vs. 186.3 ± 31.9 170.6 ± 19.4 vs. 183.4 ± 22.1 191.6 ± 34.9 vs. 204.1 ± 39.5	7.3 (5.2) 12.7 (7.3) 12.8 (7.5) 12.5 (6.5)	0.56 0.32 0.04^a^ 0.44	0.24 0.37 0.62 0.34
Padilha et al. [43]	13 14	68.9 (± 5.0) 66.7 (± 13.9)	12 weeks	CP	1 (10–15)	2 3	MF HF	35.7 ± 6.6 vs. 42.2 ± 8.0 37.1 ± 3.8 vs. 47.3 ± 4.6	6.5 (18.2) 10.2 (27.5)	≤ 0.05^b^ ≤ 0.05^b^	0.89 2.42
Padilha et al. [43]	13 14	68.9 (± 5.0)	12 weeks	LE	1 (10–15)	2 3	MF HF	41.3 ± 9.3 vs. 48.9 ± 9.1 46.1 ± 10.5 vs. 53.8 ± 12.5	7.6 (18.4) 7.7 (16.7)	≤ 0.05^b^ ≤ 0.05^b^	0.83 0.67
Padilha et al. [43]	13 14	66.7 (± 13.9)	12 weeks	BC	1 (10–15)	2 3	MF HF	17.0 ± 3.3 vs. 23.4 ± 3.8 17.7 ± 2.5 vs. 24.2 ± 3.6	6.4 (37.6) 6.5 (26.9)	≤ 0.05^b^ ≤ 0.05^b^	1.80 2.10
Hunter [39]	14 10	22.2 (± 2.2) ^c^	7 weeks	BP	2–3 (7–10)	3 4	HF HF	69.1 ± 22.08 vs. 77.3 ± 20.58 59.3 ± 25.93 vs. 69.20 ± 25.61	8.2 (11.9) 9.9 (16.7)	0.32 0.40	0.38 0.38
Hunter [39]	11 11	23.1 (± 0.9) ^d^	7 weeks	BP	2–3 (7–10)	3 4	HF HF	26.1 ± 5.97 vs. 31.3 ± 6.30 27.3 ± 7.30 vs. 36.40 ± 7.96	5.2 (19.9) 9.1 (33.3)	0.32 ≤ 0.05^b^	0.85 1.19

*Abbreviations*: *N* number of subjects, *SD* standard deviation, *reps* repetitions; *per week* number of days trained per week, *ES* effect size, *Sq* squat, *LF* low frequency (1 day per week), *MF* medium frequency (2 days per week), *HF* high frequency (≥ 3 day per week), *BP* bench press, *WGPullD* wide grip pulldown, *CP* chest press, *LP* leg press, *LumExt* lumbar extension, *CervExt* cervical extension, *LPull* lateral pulldown, *BC* bicep curl, *BExt* back extension, *LP* leg press, *KExt* knee extension, *KFlex* knee flexion, *DumBP* dumbbell bench press, *DCurl* dumbbell curl, *StCR* standing calf raise

^a^Significantly greater than prior to training (*p* ≤ 0.01)

^b^Significantly greater than prior to training (*p* ≤ 0.05)

^c^Male-only subjects

^d^Female-only subjects

**Table 4:** Characteristics of included meta-analysis studies investigating adaptations and recovery to strength training at various frequencies

Study	Design	Weekly volume equated?	*N*	Status	Sex	Age, years (mean ± SD)	Frequency	Duration	Sets	Reps	Training load	Strength outcomes
Carroll et al. [11]	RCT	Yes	17	U	M F	18.6 (± 0.9)	2 vs. 3 days week	6 weeks 9 weeks	3	4–10	4-20RM	1RM Squat
McLester et al. [17]	RAN	Yes	25	T	M F	25.5 (± 5.1)	1 vs. 3 days week	12 weeks	1–3	3–10	Periodized 75–90% 1RM	1RM Bench press, latissimus pulldown, tricep press, bicep curl, lateral raise
Brigatto et al. [22]	RAN	Yes	18	T	M	27.1(± 5.5)	1 vs. 2 days week	8 weeks	4–8	8–12	8–12 RM	1RM Bench press, 1RM squat
Gentil et al. [44]	RAN	Yes	30	U	M	(23.0 ± 3.0)	1 vs. 2 days week	10 weeks	3	8–12	8- 12RM	Maximum elbow extensor torque
Murlasits et al. [45]	RAN	No	24	U	M F	64 (± 3)	1 vs. 3 days week	8 weeks	3	6–8	75% 1RM	3 - 5RM Leg press, chest press exercises
Silva et al. [46]	RAN	No	30	U	F	68.15 (± 4.8)	2 vs. 3 days week	24 weeks	1–2	10–15	10–15 RM	Total of chest press, leg extension, biceps curl
DiFranciso-Donoghue et al. [47]	RAN	No	18	U	M F	75.2 (± 1.2)	1, 2 days week	9 weeks	1	10–15	75% 1RM	1RM Leg press, chest fly, leg curl, seated dip, leg extension, arm curl.
Lera Orsatti et al. [48]	RAN	No	30	U	F	58.3 (± 8.1)	1, 2, or 3 days week	16 weeks	1–3	8–12	60–80% 1RM	1RM Bench press, leg press, leg extension, bicep curl, tricep pulley,
Candow and Burke [49]	RAN	Yes	29	U	M F	43 (± 10.6)	2 vs. 3 days week	6 weeks	2–3	10	60–90% 1RM	1RM Bench press, 1RM squat
Schoenfeld et al. [50]	RAN	Yes	20	T	M	(23.5 ± 2.9)	1 vs. 3 days week	8 weeks	2–3	8–12	8–12 RM	1RM Bench press
Thomas and Burns [51]	RAN	Yes	19	T	M F	34.7 (± 6.9)	1 vs. 3 days week	8 weeks	3	8–12	75–85% 1RM	1RM Chest press, 1RM hack squat
Arazi and Asadi [52]	RCT	Yes	39	U	M	20.33 (± 2.1)	1 vs. 2 days week	8 weeks	1	6–12	60–80% 1RM	1RM Bench press, 1RM leg press

*Abbreviations*: *N* number, *SD* standard deviation, *Reps* repetitions, *RCT* randomised controlled trial, *U* untrained, *M* male, *F* female, *days week* days per week, *RM* repetition maximum, *1RM* 1 repetition maximum, *RAN* randomly assigned trial, *T* trained, *% 1RM* percentage of subjects one repetition maximum

**Table 5:** Pre- vs. post-strength analysis of multi-joint exercise and isolation exercise

Study	*N*	Age (mean ± SD) years	Duration	Testing modality	Sets (reps)	Days trained per week	Frequency per week	Pre- vs post-intervention kg, mean ± SD	Pre- vs post-intervention strength difference kg (% change)	*p* value (pre- vs. post)	ES
Carroll et al. [11]	6 5	18.6 (± 0.9)	9 weeks	Sq	1–3 (4–10)	2 3	MF HF	115 ± 29 vs 141 ± 29 115 ± 29 vs 152 ± 56	26 (22.6) 37 (32.2)	≤ 0.05^a b^ ≤ 0.05^a b^	0.900.83
McLester et al. [17]	9 9	25.5 (± 5.1)	12 weeks	BP	1–3 (3–10)	1 3	LF HF	75.6 ± 27.8 vs 83.6 ± 30.6 54.2 ± 25.7 vs 68.9 ± 27.5	8.01 (10.6) 14.71 (27.1)	0.57 0.26	0.27 0.55
McLester et al. [17]	9 9	25.5 (± 5.1)	12 weeks	LPull	1–3 (3–10)	1 3	LF HF	65.1 ± 20.9 vs 77.8 ± 22.9 53.8 ± 19.0 vs 63.7 ± 21.2	12.69 (19.5) 9.9 (18.4)	0.24 0.31	0.58 0.49
McLester et al. [17]	9 9	25.5 (± 5.1)	12 weeks	TriPush	1–3 (3–10)	1 3	LF HF	30.4 ± 10.3 vs 38.2 ± 13.5 21.0 ± 9.9 vs 27.7 ± 7.1	7.77 (25.5) 6.75 (32.2)	0.19 0.12	0.65 0.78
McLester et al. [17]	9 9	25.5 (± 5.1)	12 weeks	BC	1–3 (3–10)	1 3	LF HF	35.01 ± 14.8 vs 43.1 ± 16.8 25.5 ± 12.6 vs 35.2 ± 14.0	8.07 (23.1) 9.71 (38.1)	0.30 0.14	0.51 0.73
McLester et al. [17]	9 9	25.5 (± 5.1)	12 weeks	LatR	1–3 (3–10)	1 3	LF HF	22.7 ± 7.5 vs 32.3 ± 10.5 16.3 ± 6.6 vs 27.2 ± 10.6	9.63 (42.2) 10.89 (66.7)	0.04 0.02	1.05 1.23
McLester et al. [17]	9 9	25.5 (± 5.1)	12 weeks	LP	1–3 (3–10)	1 3	LF HF	200.3 ± 83.1 vs 244.9 ± 80.9 191.3 ± 96.3 vs 279.5 ± 114.3	44.6 (22.3) 88.25 (46.1)	0.23 0.10	0.54 0.83
McLester et al. [17]	9 9	25.5 (± 5.1)	12 weeks	LE	1–3 (3–10)	1 3	LF HF	62.8 ± 13.6 vs 84.2 ± 19.9 66.6 ± 15.7 vs 88.4 ± 17.1	21.41 (34.1) 21.78 (32.7)	0.02^a^ 0.01^c^	1.26 1.33
McLester et al. [17]	9 9	25.5 (± 5.1)	12 weeks	LC	1–3 (3–10)	1 3	LF HF	57.5 ± 15.5 vs 72.0 ± 10.3 43.1 ± 17.5 vs 64.0 ± 24.9	14.5 (25.2) 20.36 (47.2)	0.03^a^ 0.06	1.10 0.97
McLester et al. [17]	9 9	25.5 (± 5.1)	12 weeks	CR	1–3 (3–10)	1 3	LF HF	178.0 ± 24.2 vs 215.1 ± 15.3 131.7 ± 46.0 vs 163.0 ± 42.5	37.1 (20.8) 31.29 (23.8)	0.001^a^ 0.15	1.83 0.71
Brigatto et al. [22]	10 10	28.6 (± 5.6) 5.5 (± 5.1)	8 weeks	Sq	8 (8–12)	1 2	LF MF	128.5 ± 18.6 vs 148.6 ± 21.7 121.1 ± 17.2 vs 140.6 ± 25.4	20.1 (13.5) 19.5 (13.9)	≤ 0.05^a^ ≤ 0.05^a^	0.99 0.90
Brigatto et al. [22]	10 10	28.6 (± 5.6) 5.5 (± 5.1)	8 weeks	BP	8 (8–12)	1 2	LF MF	95.7 ± 14.5 vs 103.5 ± 12.9 92.6 ± 14.3 vs 100.4 ± 13.3	7.8 (8.2) 7.8 (8.4)	≤ 0.05^a^ ≤ 0.05^a^	0.57 0.56
Gentil et al. [44]	15 15	23.0 (± 3.0)	10 weeks	EFlex	3 (8–12)	1 2	LF MF	50.77 ± 9.26 vs 54.15 ± 10.79 48.99 ± 11.52 vs 55.29 ± 10.24	3.38 (6.66) 6.3 (12.86)	≤ 0.05^a^ ≤ 0.05^a^	0.34 0.58
Murlasits et al. [45]	13 11	64 (± 3)	8 weeks	CP	3 (8)	2 3	MF HF	43.1 ± 20.2 vs 51.8 ± 24.7 40.8 ± 16.9 vs 49.1 ± 20.8	8.7 (20.84) 8.3 (20.18)	0.01^c^ 0.01^c^	0.39 0.44
Murlasits et al. [45]	13 11	64 (± 3)	8 weeks	LP	3 (8)	2 3	MF HF	158.9 ± 49.6 vs 193 ± 57.7 157.2 ± 55.6 vs 192.3 ± 62.3	34.1 (22.34) 35.1 (28.12)	0.01^c^ 0.01^c^	0.63 0.59
Silva et al. [46]	17 13	68.2 (± 4.8)	24 weeks	CP, LE, BC	1–2 (10–15)	2 3	MF HF	100.6 ± 13.4 vs 117.4 ± 17.6 110.9 ± 18.0 vs 129.5 ± 19.5	16.8 (16.8) 18.6 (18.9)	≤ 0.05^a^ ≤ 0.05^a^	1.07 0.99
DiFranciso-Donoghue et al. [47]	9 9	75.2 (± 1.2)	9 weeks	LP	1 (10–15)	1 2	LF MF	35.6 ± 13.5 vs 46.2 ± 15.3 37.6 ± 10.2 vs 51.5 ± 12.0	10.6 (29.8) 13.9 (40.0)	≤ 0.01^c^ ≤ 0.01^c^	0.73 1.25
DiFranciso-Donoghue et al. [47]	9 9	75.2 (± 1.2)	9 weeks	LE	1 (10–15)	1 2	LF MF	34.3 ± 12.0 vs 42.9 ± 10.5 29.2 ± 10.8 vs 40.7 ± 16.2	8.6 (25.1) 11.5 (39.4)	≤ 0.01^c^ ≤ 0.01^c^	0.76 0.84
DiFranciso-Donoghue et al. [47]	9 9	75.2 (± 1.2)	9 weeks	LC	1 (10–15)	1 2	LF MF	21.0 ± 8.1 vs 27.8 ± 7.5 21.2 ± 7.8 vs 30.1 ± 14.4	6.8 (32.4) 8.9 (42.0)	≤ 0.01^c^ ≤ 0.01^c^	0.87 0.77
DiFranciso-Donoghue et al. [47]	9 9	75.2 (± 1.2)	9 weeks	CF	1 (10–15)	1 2	LF MF	12.8 ± 8.1 vs 18.4 ± 9.3 14.4 ± 9.3 vs 21.9 ± 12.6	5.6 (43.8) 7.5 (52.0)	≤ 0.01^c^ ≤ 0.01^c^	0.64 0.68
DiFranciso-Donoghue et al. [47]	9 9	75.2 (± 1.2)	9 weeks	ArmC	1 (10–15)	1 2	LF MF	12.1 ± 6.9 vs 15.9 ± 6.6 9.7 ± 7.5 vs 14.4 ± 9.3	3.8 (31.4) 4.7 (48.4)	≤ 0.01^c^ ≤ 0.01^c^	0.56 0.56
DiFranciso-Donoghue et al. [47]	9 9	75.2 (± 1.2)	9 weeks	SDip	1 (10–15)	1 2	LF MF	36.4 ± 11.4 vs 44.7 ± 9.6 41.3 ± 12.9 vs 48.9 ± 12.6	8.3 (22.8) 7.6 (18.4)	≤ 0.01^c^ ≤ 0.01^c^	0.79 0.60
Lera Orsatti et al. [48]	9 11 10	58.3 (± 8.1)	16 weeks	BP	3 (8–15)	1 2 3	LF MF HF	30.8 ± 6.5 vs 36.5 ± 4.3 25.5 ± 6.0 vs 35.4 ± 8.2 31.7 ± 7.6 vs 45.2 ± 12.5	5.7 (18.5) 9.9 (38.8) 13.5 (42.6)	≤ 0.01^c^ ≤ 0.01^c^ ≤ 0.0 ^c b^	1.03 1.38 1.31
Lera Orsatti et al. [48]	9 11 10	58.3 (± 8.1)	16 weeks	LP	3 (8–15)	1 2 3	LF MF HF	30.5 ± 5.6 vs 36.4 ± 2.2 26.3 ± 5.6 vs 30.4 ± 6.1 30.1 ± 4.4 vs 35.0 ± 7.7	5.9 (19.3) 4.1 (15.6) 4.9 (16.3)	≤ 0.01^c^ ≤ 0.01^c^ ≤ 0.01 ^c^	1.39 0.70 0.78
Lera Orsatti et al. [48]	9 11 10	58.3 (± 8.1)	16 weeks	LE	3 (8–15)	1 2 3	LF MF HF	27.7 ± 2.1 vs 30.7 ± 5.0 25.5 ± 4.5 vs 29.1 ± 4.8 25.9 ± 5.3 vs 33.1 ± 4.4	3.0 (10.8) 3.6 (14.1) 7.2 (27.8)	≤ 0.01^c^ ≤ 0.01^c^ ≤ 0.01^c^	0.78 0.77 1.48
Lera Orsatti et al. [48]	9 11 10	58.3 (± 8.1)	16 weeks	BC	3 (8–15)	1 2 3	LF MF HF	19.0 ± 4.0 vs 21.1 ± 2.8 17.4 ± 4.8 vs 19.4 ± 4.4 20.3 ± 3.1 vs 22.7 ± 3.2	2.1 (11.1) 2.0 (11.5) 2.4 (11.8)	≤ 0.01^c^ ≤ 0.01^c^ ≤ 0.01^c^	0.61 0.43 0.76
Lera Orsatti et al. [48]	9 11 10	58.3 (± 8.1)	16 weeks	TriPull	3 (8–15)	1 2 3	LF MF HF	14.3 ± 2.7 vs 16.5 ± 2.6 12.8 ± 2.7 vs 15.3 ± 3.3 14.9 ± 2.6 vs 17.3 ± 3.1	2.2 (15.4) 2.5 (19.5) 2.4 (16.1)	≤ 0.01^c^ ≤ 0.01^c^ ≤ 0.01^c^	0.83 0.83 0.84
Candow and Burke [49]	15 14	42.5 (± 15.5)	6 weeks	Sq	2–3 (10)	2 3	MF HF	39 ± 3.4 vs 46.17 ± 10.2 37.1 ± 3.8 vs 48.20 ± 10.2	7.2 (18.4) 11.1 (29.9)	≤ 0.05^a^ ≤ 0.05^a^	0.94 1.44
Candow and Burke [49] excluded	15 14	42.5 (± 15.5)	6 weeks	BP	2–3 (10)	2 3	MF HF	109.4 ± 8.5 vs 140.22 ± 25.1 112.1 ± 8.7 vs 143.46 ± 23.4	30.8 (28.2) 31.4 (28)	≤ 0.05^a^ ≤ 0.05^a^	1.64 1.78
Schoenfeld et al. [50]	10 9	23.5 (± 2.9)	4 weeks	BP	3 (8–12)	1 3	LF HF	92.9 ± 21.5 vs 99.4 ± 21 97.0 ± 20 vs 107.5 ± 17.9	6.46 (7.0) 10.5 (10.8)	≤ 0.01^a^ ≤ 0.05^a^	0.31 0.55
Schoenfeld et al. [50]	10 9	23.5 (± 2.9)	4 weeks	Sq	3 (8–12)	1 3	LF HF	114.8 ± 31.8 vs 127.1 ± 30.6 122.7 ± 29.8 vs 137.6 ± 27.5	12.27 (10.7) 14.9 (12.1)	≤ 0.01^a^ ≤ 0.05^a^	0.39 0.52
Thomas and Burns [51]	10 9	34.2 (± 11) 35.1 (± 6.9)	8 weeks	HS	3 (8–12)	1 3	LF HF	90.2 ± 41.5 vs 112.0 ± 55.4 96.8 ± 40.31 vs 116.9 ± 55.3	21.83 (24.2) 20.16 (20.8)	≤ 0.05^a^ ≤ 0.05^a^	0.45 0.42
Thomas and Burns [51]	10 9	34.2 (± 11) 35.1 (± 6.9)	8 weeks	CP	3 (8–12)	1 3	LF HF	78.6 ± 40.78 vs 84.4 ± 58.3 84.8 ± 31.41 vs 93.9 ± 41.5	5.8 (7.4) 9.07 (10.7)	≤ 0.05^a^ ≤ 0.05^a^	0.12 0.25
Arazi and Asadi [52]	10 10 9	20.20 (± 1.9) 20.40 (± 2.3) 20.33 (± 1.8)	8 weeks	BP	1 (6–12)	1 2 3	LF MF HF	47.5 ± 10.6 vs 52.5 ± 5.2 43.1 ± 13.2 vs 49.5 ± 7.8 45.1 ± 10.7 vs 51.2 ± 6.6	5.0 (10.6) 6.4 (14.8) 6.1 (13.6)	≤ 0.05^a^ ≤ 0.05^a^ ≤ 0.05^a^	0.60 0.59 0.69
Arazi and Asadi [52]	10 10 9	20.20 (± 1.9) 20.40 (± 2.3) 20.33 (± 1.8)	8 weeks	LP	1 (6–12)	1 2 3	LF MF HF	87.5 ± 17.5 vs 95.0 ± 19.3 87.0 ± 22.9 vs 94.5 ± 21.5 94.0 ± 19.4 vs 100.7 ± 19.2	7.5 (8.6) 7.5 (8.6) 6.7 (7.1)	≤ 0.05^a^ ≤ 0.05 ^a^ ≤ 0.05^a^	0.41 0.34 0.35

*Abbreviations*: *N* number of subjects, *SD* standard deviation, *reps* repetitions, *per week* number of days trained per week, *ES* effect size, *Sq* squat, *BP* bench press, *MF* medium frequency (2 days per week), *HF* high frequency (≥ 3 day per week), *LPull* lateral pulldown, *LF* low frequency (1 day per week), *TriPush* tricep pushdown, *BC* bicep curl, *LatR* lateral raise, *LP* leg press, *LE* leg extension, *LC* leg curl, *CR* calf raise, *EFlex* elbow flexion, *CF* chest fly, *ArmC* arm curl, *SDip* seated dip, *TriPull* tricep pulldown, *HS* hack squat

^a^Significantly greater than prior to training (*p* ≤ 0.05)

^b^Significant differences from corresponding groups-exercise values (*p* ≤ 0.05)

^c^Significantly greater than prior to training (*p* ≤ 0.01)

### Sensitivity Analysis

**Fig. 2:**
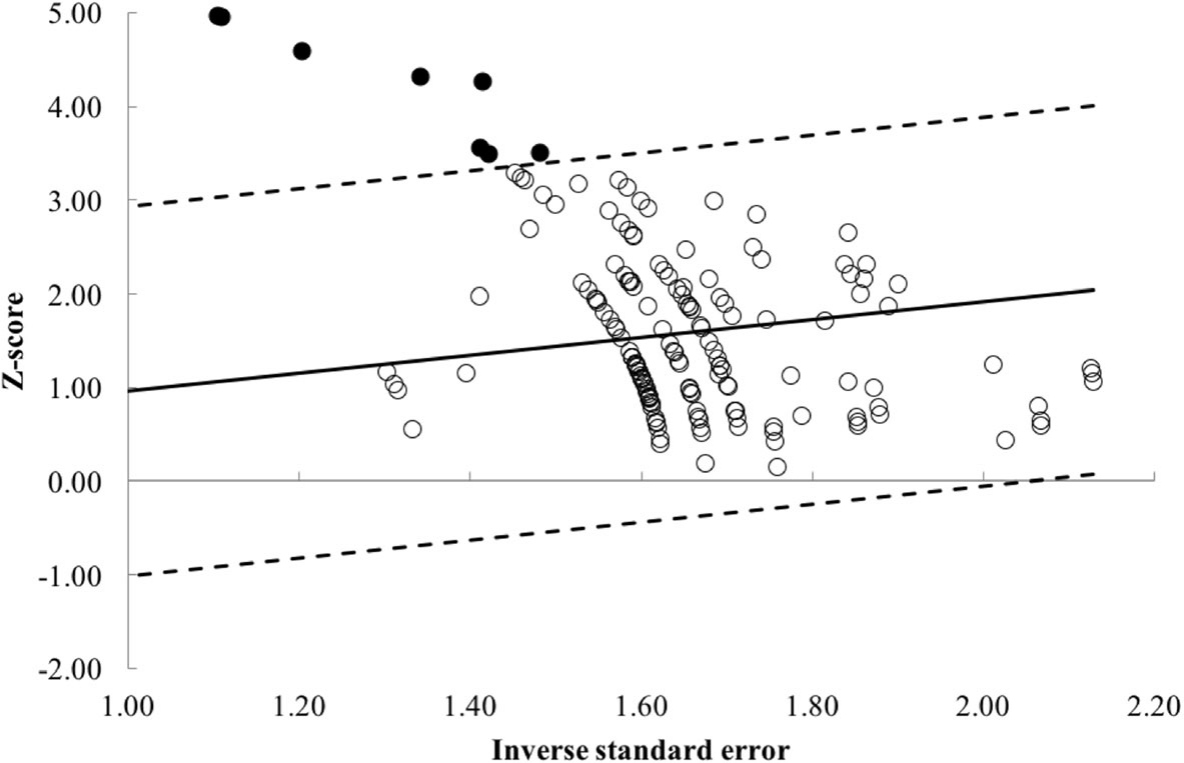
Galbraith plot used to examine study heterogeneity (pre- vs. post-strength change). Each dot represents one pre- vs. post-study data. Seven pre- vs. post-study data identified as outliers (solid filled black circles)

**Fig. 3:**
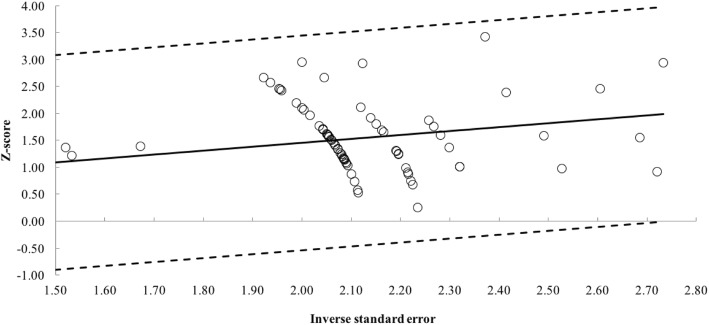
Galbraith plot with the removal of seven pre- vs. post-intervention study outliers [20, 21, 23, 40–43]. Each open circle represents one pre- vs.-post-intervention study datum

Further examination of study heterogeneity with Galbraith plots used to identify any potential outliers (Figs. 2 and 3) revealed that pre- vs. post data were influential [20, 21, 23, 40–43]. Removal changed the statistical outcome of weekly RT frequency on strength gain (Fig. 3). In the assessment of publication bias, moderate asymmetry was initially observed in the funnel plot of multi-joint and isolation data. Duval and Tweedie’s [33] trim and fill correction procedure was used. This method shifted the overall ES from 0.98 to 0.72, with a significant effect on *p* value (*p =* 0.001). No apparent asymmetry was exposed via the funnel plot, once data point outliers were removed.

### RT Study Characteristics

Following review and sensitivity procedures, the full text of 12 articles [11, 17, 22, 44–52] was deemed to meet inclusion criteria (Table 1). Articles included in the analysis had dates ranging from 1998 to 2018. The experimental design of included studies had a random assignment of treatment conditions (RAN) and randomised control groups (RCT). The subjects training status included in the 12 studies was trained (*n* = 4) and untrained (*n* = 8). In total, 12 studies provided data on a total of 299 subjects (Table 4). The mean age of the subjects was 40 (± 19.9 years.). Assigned cohorts consisted of male (36%), female (20%)-only groups, and mixed-sex studies (44%) were included in the analysis. The training length ranged from 8 to 24 weeks (mean = 10.5 (± 4.75 weeks), frequency ranged from 1 to 3 days week^− 1^, and the exercise repetition ranged from 3 to 15 repetitions. The number of sets specified ranged from 1 to 8 sets.

### Effects of Weekly Training Frequency on Multi-Joint and Isolation Combined

Pre- to post-strength measures were assessed via the meta-analytic procedure for all included studies. This was followed by subgroup analysis with multi-joint and isolation exercises combined into separate sub-group analysis. Due to the potential of significant heterogeneity of data, a random effects model was incorporated into each strength measure with *I*^2^ used to assess heterogeneity.

### Effects of Frequency on Combined Multi-Joint and Isolation Exercise

**Fig. 4:**
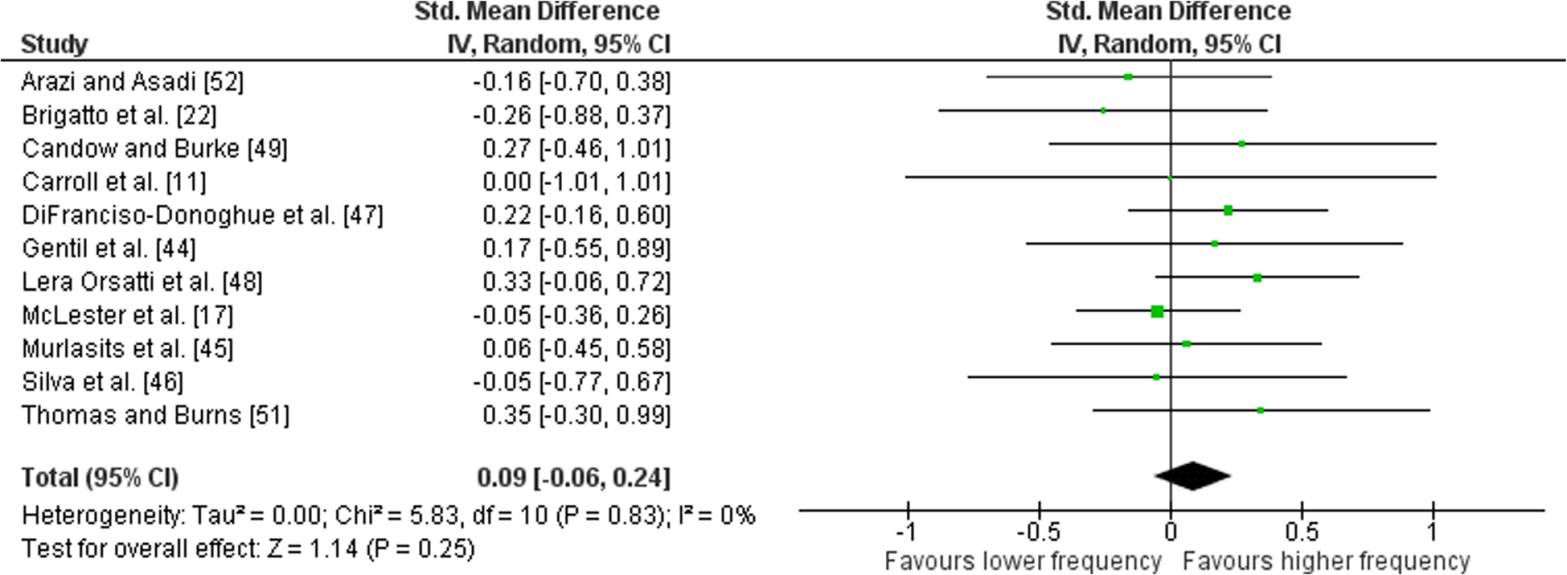
Lower vs. higher weekly training frequency. Pre- vs. post-mean ES strength difference on multi-joint and isolation exercise. The vertical line indicates the overall estimate of combined multi-joint and isolation studies pre- vs. post-mean ES strength difference. Horizontal lines indicate 95% CI, squares estimates, whereas square size is proportional to sample size and rhombs’ meta-analytically pooled estimates

**Fig. 5:**
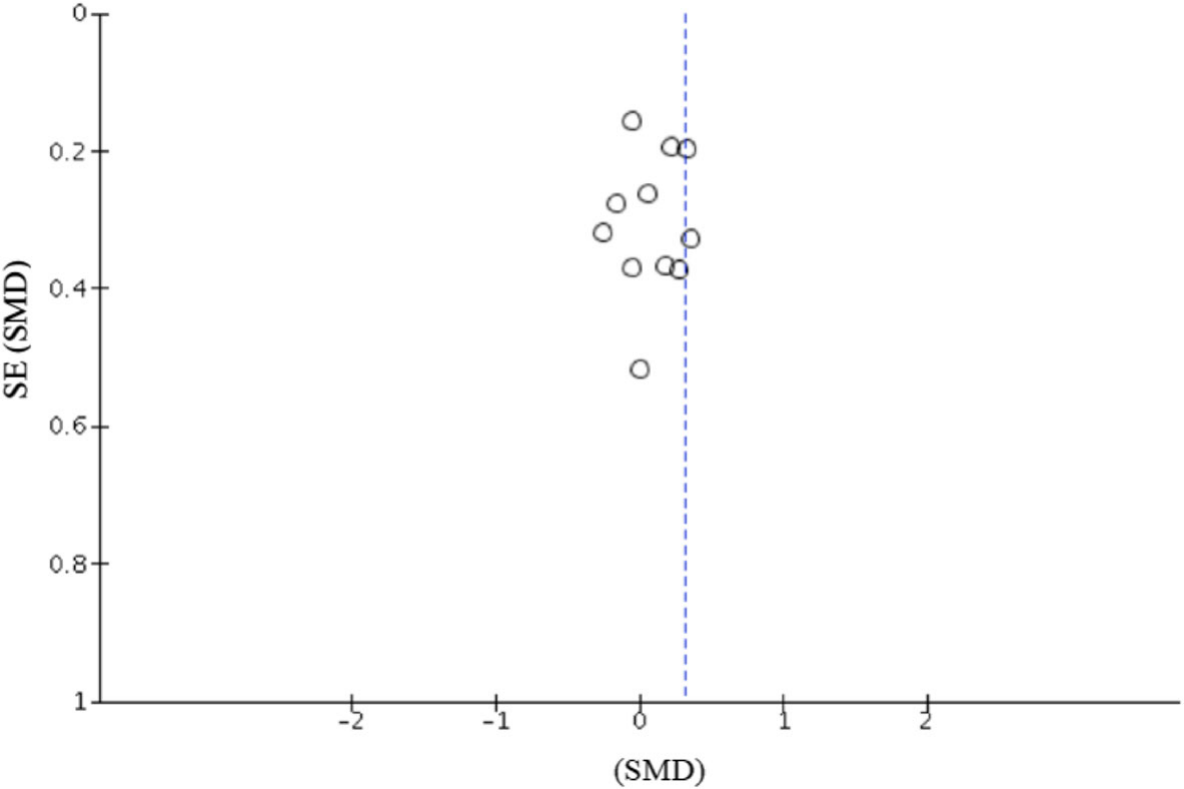
Funnel plot of standard error (SE) by the standard mean difference (SMD) for assessment of publication bias with included resistance training frequency studies (Schoenfeld et al. [50] data excluded). Each open circle denotes a study included in the meta-analysis. The blue dashed vertical line represents the overall effect calculated with the random-effects model

Outcomes for weekly training frequency categorised as lower or higher frequency are presented in the forest plot (Fig. 4). The forest plot contains the mean ES and corresponding CIs for strength gain separated for interventions featuring lower and higher frequency, as well as the overall effect test and heterogeneity analysis. The pooled mean ES estimates of multi-joint and isolation data (Table 5) comprised of 74 treatment groups from 12 studies [11, 17, 22, 44–52]. There was significant heterogeneity detected in the 12 studies (*I*^2^ = 82%), with Schoenfeld et al. [50] identified as being influential. Removal of the Schoenfeld et al. [50] study resulted in no heterogeneity being detected in the other 11 studies (Fig. 5). When a random effect analysis was applied, a small effect was observed for multi-joint and isolation weekly training frequency (ES 0.09; 95% CI − 0.06–0.24). Pre- to post-intervention strength gain was marginally greater with HF compared to LF (ES difference 0.07); however, the effect was not statistically significant (*p* = 0.25). The mean for lower frequency was 0.71 (95% CI 0.56–0.86). The mean ES for higher frequency was 0.78 (95% CI 0.60–0.96).

**Fig. 6:**
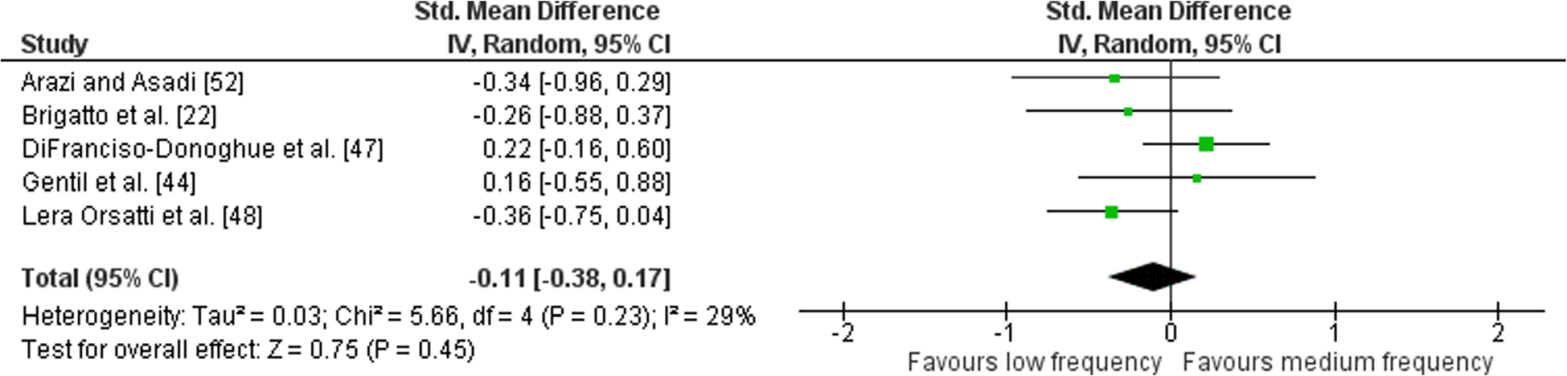
Low vs. medium weekly training frequency. Pre- vs. post-mean ES strength difference on multi-joint and isolation exercises. The vertical line indicates the overall estimate of combined multi-joint and isolation studies pre- vs. post-mean ES strength difference. Horizontal lines indicate 95% CI, squares estimates, whereas square size is proportional to sample size, and rhombs’ meta-analytically pooled estimates

Outcomes for weekly training frequency categorised as LF or MF within each study are shown in Fig. 6. Low heterogeneity was detected in the five studies (*I*^2^ = 29%). When a random effect analysis was applied, a trivial effect was observed for multi-joint and isolation weekly training frequency (ES − 0.11; 95% CI − 0.38–0.17). Pre- to post-intervention strength gain was similar when LF was compared to MF (ES difference 0.02) with no statistical significance between RT frequencies (*p* = 0.45). The mean for LF was 0.68 (95% CI 0.55–0.80). The mean ES for MF was 0.66 (95% CI 0.45–0.86).

**Fig. 7:**
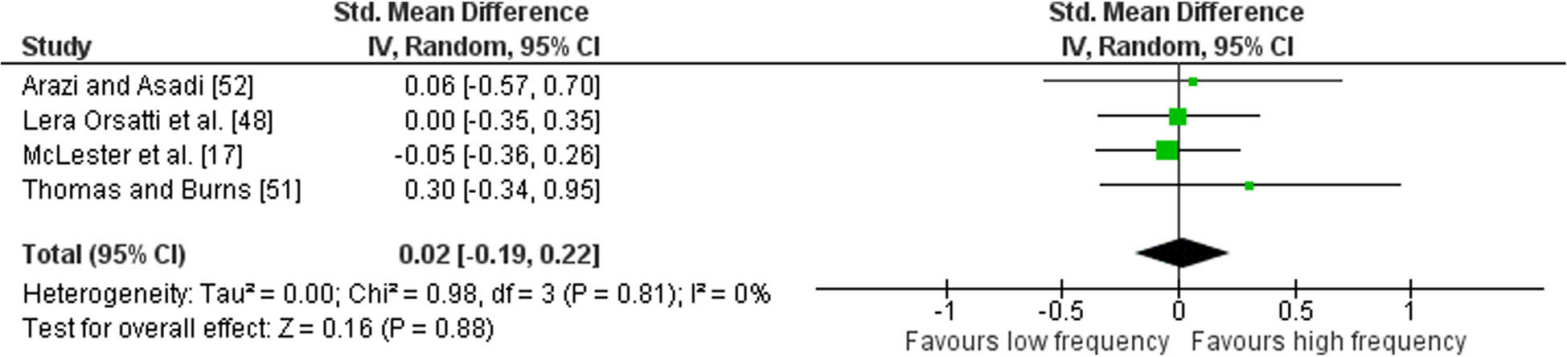
Low vs. high weekly training frequency. Pre- vs. post-mean ES strength difference on multi-joint and isolation exercise. The vertical line indicates the overall estimate of combined multi-joint and isolation studies pre- vs. post-mean ES strength difference. Horizontal lines indicate 95% CI, squares estimates, whereas square size is proportional to sample size, and rhombs’ meta-analytically pooled estimates

Outcomes for weekly training frequency categorised as LF or HF within each study are shown in Fig. 7. No heterogeneity was detected in the four studies (*I*^2^ = 0%). When a random effect analysis was applied, a trivial effect was observed for multi-joint and isolation weekly training frequency (ES 0.02; 95% CI − 0.19–0.22). Pre- to post-intervention strength gain was similar when LF was compared to HF (ES difference 0.01) with no statistical significance between RT frequencies (*p* = 0.88). The mean for LF was 0.65 (95% CI 0.35–0.95). The mean ES for HF was 0.66 (95% CI 0.39–0.93).

**Fig. 8:**
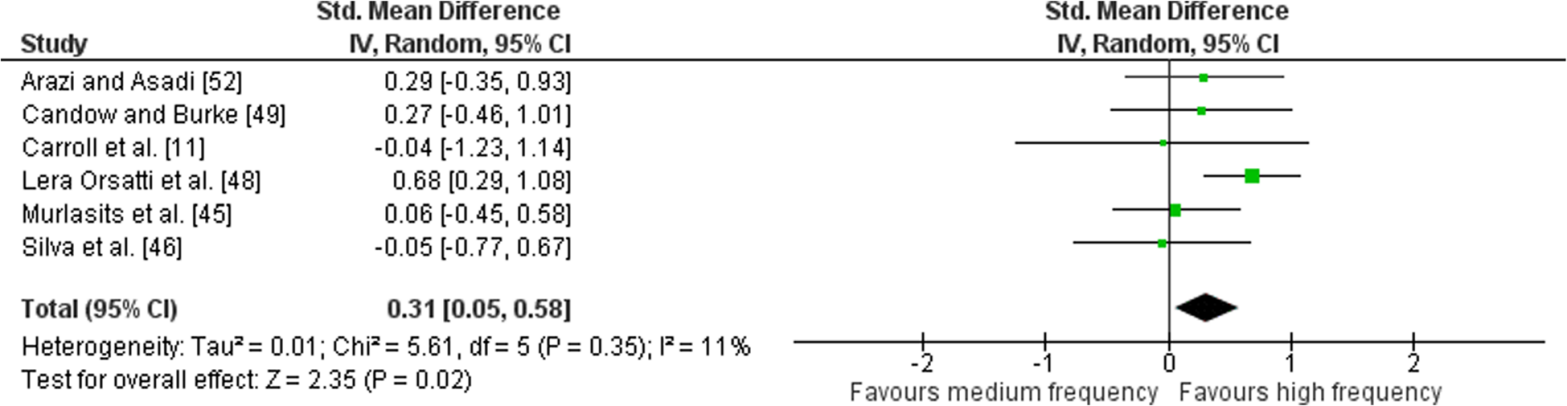
Medium vs. high weekly training frequency. Pre- vs. post-mean ES strength difference on multi-joint and isolation exercise. The vertical line indicates the overall estimate of combined multi-joint and isolation studies pre- vs. post-mean ES strength difference. Horizontal lines indicate 95% CI, squares estimates, whereas square size is proportional to sample size, and rhombs’ meta-analytically pooled estimates

Outcomes for weekly training frequency categorised as MF or HF within each study are shown in Fig. 8. Low heterogeneity was detected in the six studies (*I*^2^ = 11%). When a random effect analysis was applied, a small effect was observed for multi-joint and isolation weekly training frequency (ES 0.31; 95% CI 0.05–0.58). Pre- to post-intervention strength gain was marginally greater with HF compared to MF (ES difference 0.09) with the effect statistically significant (*p* = 0.02). The mean for HF was 0.88 (95% CI 0.61–1.17). The mean ES for MF was 0.79 (95% CI 0.59–0.98).

### Effects of Frequency on Volume-Equated Combined Multi-Joint and Isolation Exercise

**Fig. 9:**
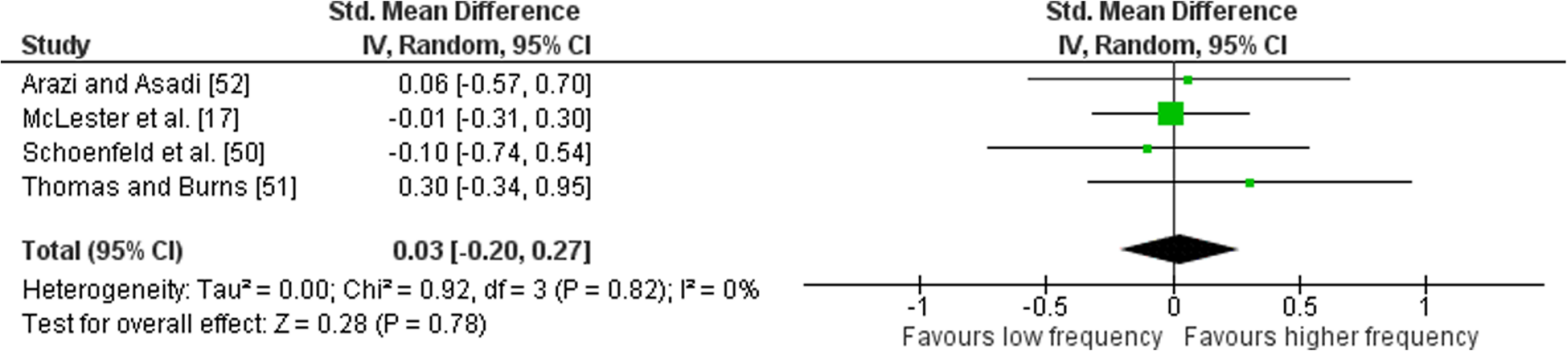
Low vs. high weekly training frequency. Pre- vs. post-mean ES strength difference on volume-equated multi-joint and isolation exercise. The vertical line indicates the overall estimate of combined multi-joint and isolation studies pre- vs. post-mean ES strength difference. Horizontal lines indicate 95% CI, squares estimates, whereas square size is proportional to sample size, and rhombs’ meta-analytically pooled estimates

Outcomes for volume-equated weekly training frequency categorised as either LF or HF are shown in Fig. 9. The forest plot contains the mean ES and corresponding CIs for strength gain separated for interventions featuring LF and HF as well as the overall effect test and heterogeneity analysis. The pooled mean ES estimates of volume-equated multi-joint and isolation data comprised of 28 treatment groups from four studies [17, 50–52]. No heterogeneity was detected in the four studies (*I*^2^ = 0%). When a random effect analysis was applied, a trivial effect was observed for multi-joint and isolation weekly training frequency (ES 0.03; 95% CI − 0.20–0.27). Pre- to post-intervention strength gain was similar when LF was compared to HF (ES difference 0.01) with no statistical significance between RT frequencies (*p* = 0.78). The mean for LF was 0.54 (95% CI 0.30–0.77). The mean ES for HF was 0.55 (95% CI 0.33–0.76). Subgroup examination of LF vs. MF or MF vs. HF pre- to post-intervention strength differences was not feasible due to limited study data.

### Effects of Frequency on Upper Body Exercise

**Fig. 10:**
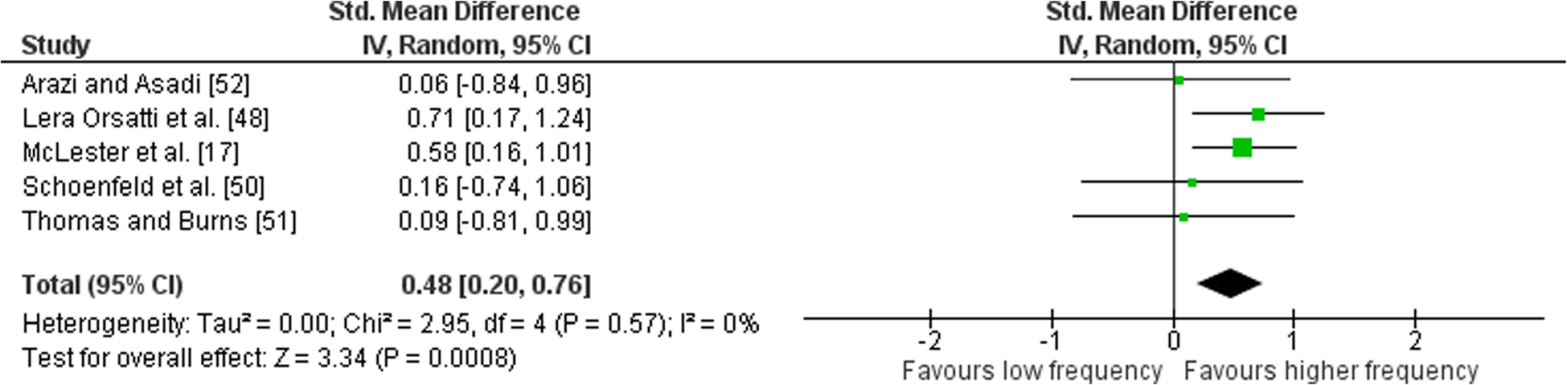
Low vs. high weekly training frequency. Pre- vs. post-mean ES strength difference on upper body exercise. The vertical line indicates the overall estimate of combined multi-joint and isolation studies pre- vs. post-mean ES strength difference. Horizontal lines indicate 95% CI, squares estimates, whereas square size is proportional to sample size, and rhombs’ meta-analytically pooled estimates

Outcomes for weekly training frequency categorised as LF or HF for upper body multi-joint and isolation exercises are shown in the forest plot (Fig. 10). The pooled mean ES estimates of the upper body combined exercises comprised of 16 treatment groups from five studies [17, 48, 50–52]. No heterogeneity was detected in the five studies (I^2^ = 0%). When a random-effects analysis was applied, a small effect was observed (ES 0.48; 95% CI 0.20–0.76). Pre- to post-intervention strength gain was greater when HF was compared with LF (ES difference 0.15) with the effect statistically significant (*p* < 0.01). The mean ES for LF was 0.49 (95% CI 0.25–0.73). The mean ES for HF was 0.64 (95% CI 0.341–0.88). Examination of MF vs. HF and LF vs. MF pre- to post-intervention strength differences was not feasible due to limited study data.

**Fig. 11:**
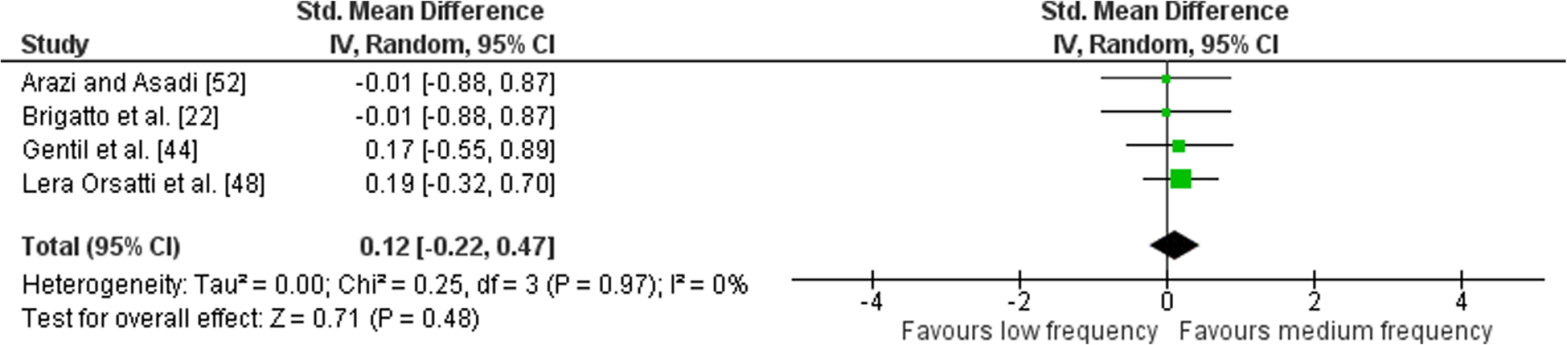
Low vs. medium weekly training frequency. Pre- vs. post-mean ES strength difference on upper body exercise. The vertical line indicates the overall estimate of combined multi-joint and isolation studies pre- vs. post-mean ES strength difference. Horizontal lines indicate 95% CI, squares estimates, whereas square size is proportional to sample size, and rhombs’ meta-analytically pooled estimates

Outcomes for weekly training frequency categorised as LF or MF for upper body exercises are shown in the forest plot (Fig. 11). The pooled mean ES estimates of the upper body combined exercises comprised of 16 treatment groups from five studies [22, 44, 47, 48, 52]. Heterogeneity was detected in the five studies (*I*^2^ = 60%), with DiFranciso-Donoghue et al. [47] identified as being influential. Removal of DiFranciso-Donoghue et al. [47] study resulted in no heterogeneity being detected in the other four studies (*I*^2^ = 0%). When a random-effects analysis was applied, a trivial effect was observed (ES 0.12; 95% CI − 0.22–0.47). Pre- to post-intervention strength gain was marginally greater when MF was compared with LF (ES difference 0.07); however, the effect was not significant (*p* = 0.48). The mean ES for LF was 0.58 (95% CI 0.39–0.77). The mean ES for MF was 0.65 (95% CI 0.50–0.80). Examination of MF vs. HF pre- to post-intervention strength differences was not feasible due to limited study data.

### Effects of Frequency on Lower Body Exercise

**Fig. 12:**
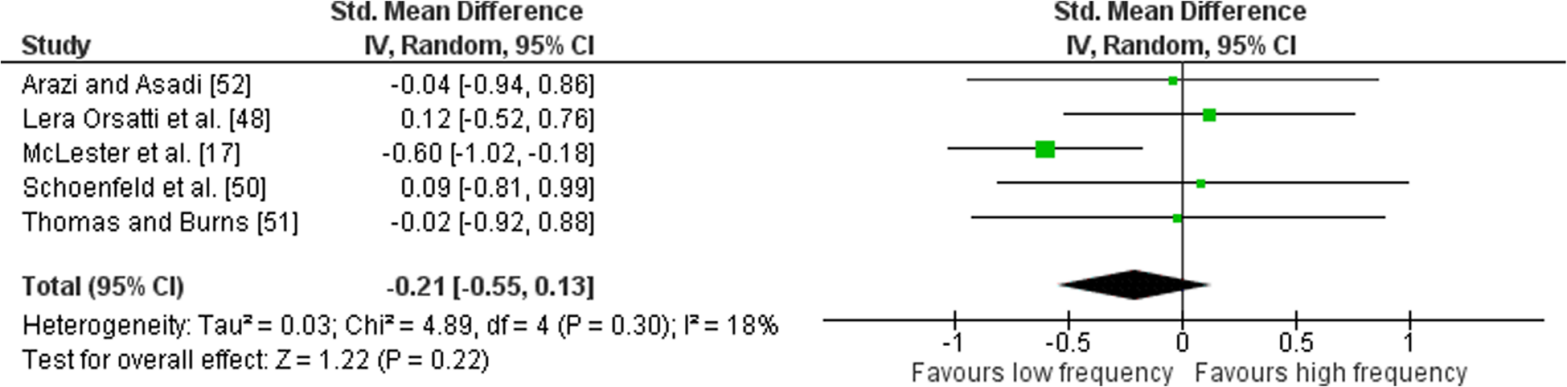
Low vs. high weekly training frequency. Pre- vs. post-mean ES strength difference on lower body exercise. The vertical line indicates the overall estimate of combined multi-joint and isolation studies pre- vs. post-mean ES strength difference. Horizontal lines indicate 95% CI, squares estimates, whereas square size is proportional to sample size, and rhombs’ meta-analytically pooled estimates

Outcomes for weekly training frequency categorised as LF or HF for lower body exercises are shown in the forest plot (Fig. 12). The pooled mean ES estimates of the lower body exercises comprised of 18 treatment groups from five  studies [17, 48, 50–52]. Low heterogeneity was detected in the five studies (*I*^2^ = 18%). When a random-effects analysis was applied, a small effect was observed (ES − 0.21; 95% CI − 0.55–0.13). Pre- to post-intervention strength gain was similar when HF was compared with LF (ES difference 0.02) with no statistical significance (*p* = 0.22). The mean ES for LF was 0.70 (95% CI 0.36–1.05). The mean ES for HF was 0.68 (95% CI 0.37–0.98). Examination of MF vs. HF and LF vs. MF pre- to post-intervention strength differences was not feasible due to limited study data.

**Fig. 13:**
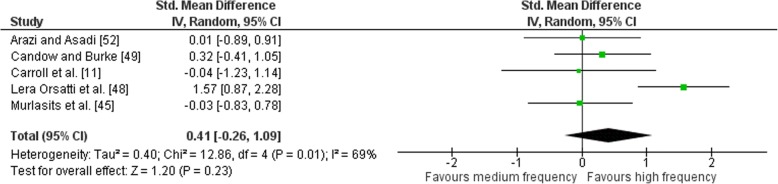
Medium vs. high weekly training frequency. Pre- vs. post-mean ES strength difference on lower body exercise. The vertical line indicates the overall estimate of combined multi-joint and isolation studies pre- vs. post-mean ES strength difference. Horizontal lines indicate 95% CI, squares estimates, whereas square size is proportional to sample size, and rhombs’ meta-analytically pooled estimates

Outcomes for weekly training frequency categorised as MF or HF for lower body exercises are shown in the forest plot (Fig. 13). The pooled mean ES estimates of the lower body exercises comprised of 12 treatment groups from five studies [11, 45, 48, 49, 52]. Moderate heterogeneity was detected in the five studies (*I*^2^ = 69%). When a random-effects analysis was applied, a small effect was observed (ES 0.41; 95% CI − 0.26–1.09). Pre- to post-intervention strength gain was greater when HF was compared with MF (ES difference 0.16); however, the effect was not significant (*p* = 0.23). The mean ES for HF was 0.87 (95% CI 0.49–1.25). The mean ES for MF was 0.71 (95% CI 0.50–0.92). Examination of MF vs. HF and LF vs. MF pre- to post-intervention strength differences was not feasible due to limited study data.

### Effects of Weekly Training Frequency on Isolation-Only Exercise

**Fig. 14:**
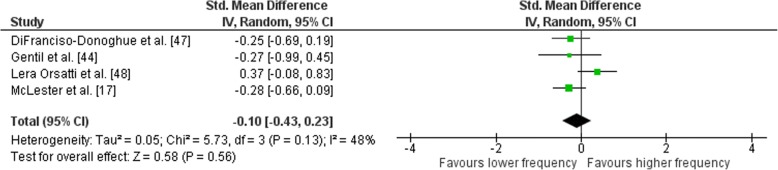
Lower vs. higher weekly training frequency. Pre- vs. post-mean ES strength difference on isolation exercise. The vertical line indicates the overall estimate of combined multi-joint and isolation studies pre- vs. post-mean ES strength difference. Horizontal lines indicate 95% CI, squares estimates, whereas square size is proportional to sample size, and rhombs’ meta-analytically pooled estimates

Outcomes for weekly training frequency categorised as lower or higher frequency for isolation exercises are displayed in the forest plot (Fig. 14). The pooled mean ES estimates of isolation exercises comprised of 30 treatment groups from four studies [17, 44, 47, 48]. There was moderate heterogeneity detected in the four studies (*I*^2^ =  48%). When a random-effects analysis was applied, a trivial effect was observed (ES − 0.10; 95% CI − 0.43–0.23). Pre- to post-intervention strength gain was marginally greater with LF compared with HF (ES difference 0.11) however the effect was not statistically significant (*p* = 0.56). The mean ES for LF was 0.88 (95% CI 0.71–1.04). The mean ES for HF was 0.77 (95% CI 0.61–0.93). Subgroup examination of LF vs. HF, MF vs. HF, and LF vs MF pre- to post-intervention strength differences was not feasible due to limited study data.

## Discussion

The purpose of this paper was to conduct a meta-analysis that (1) quantified the effects of low (LF; 1 day week^− 1^), medium (MF; 2 days week^− 1^), or high (HF; ≥ 3 days week^− 1^) RT frequency on muscular strength per exercise; (2) examined the effects of different RT frequency on one repetition maximum (1RM) strength gain profiles (multi-joint exercises and single joint exercises); (3) examined the effects of different RT frequency on 1RM strength gain when RT volume is equated; and (4) examined the effects of different RT frequency on 1RM strength gains on upper and lower body.

This paper is the second systematic review that compares different RT frequencies and provides evidence from additional studies that investigates a graded dose-response relationship where strength gains are developed following increased training frequency. Furthermore, results from this meta-analysis highlight the need for further research exploring methods used in professional practice. Although this meta-analysis endeavoured to include research papers from high-quality sources, the number of suitable studies was small and there remained differences in design and control among included studies. This consequently produces issues that may influence data reliability including the low statistical power due to small pooled sample sizes.

### Recommendations on Weekly Training Frequency

The existing evidence on the effect of weekly RT frequency has on strength development has been produced from limited and substantiated scientific evidence. Exercise physiology literature suggests that beginners train 2 to 3 days week^− 1^ and that more experienced subjects engage in more frequent training [53]. The ACSM position stand [10] cites 16 RT studies that support their frequency recommendations for strength development; for untrained [17, 29, 53–56]; intermediate [16, 17, 49, 53, 57]; and well-trained subjects [38, 53, 58]. The position stand [10] recommends that novices (those with no RT experience or have not trained for several years) train the entire body 2 to 3 days week^− 1^. For intermediate subjects, a similar frequency of 2 to 3 days week^− 1^ total-body workouts or split routines (upper body/lower body) to provide a higher volume of exercise. The RT frequency of 4 to 5 days week^− 1^ for advanced weightlifters, powerlifters, and bodybuilders has been suggested for strength development.

A recent meta-analysis by Grgic et al. [19] compared different RT weekly frequencies (1, 2, 3, and ≥ 4 days week^− 1^) on muscular strength gains. The results of their analysis indicated a significant effect (*p* = 0.03) on muscular strength was achieved when weekly RT frequency was increased. The ES increased with each additional weekly RT session from 0.74, 0.82, 0.93, and 1.08 when training 1, 2, 3, and ≥ 4 days week^− 1^. Subgroup analysis for 1RM strength test on multi-joint exercise showed a significant effect (*p* ≤ 0.001), but not single-joint exercise (*p* = 0.324). Analysis of upper body revealed a significant effect of frequency (*p* = 0.004), but not the lower body (*p* = 0.07) on strength gains. A significant effect of training frequency was reported among young adults (*p* = 0.024) but not the middle or older aged adults (*p* = 0.093). In addition, subgroup analysis for sex identified a significant difference of RT frequency in females (*p* = 0.03), but not males (*p* = 0.19). However, when subgroup analysis was performed on volume-equated studies, no significant effect (*p* = 0.421) of RT training frequency on muscular strength gains was observed.

The results of this analysis (without accounting for training volume) cannot fully support the findings of Grgic et al. [19] regarding the contention that increased weekly training frequency is superior to lower weekly frequency. In this current review when combining multi-joint and isolation exercises, a similar strength gain relationship was observed with HF compared to LF. Analysis of upper and lower body pre- to post-strength was comparable when performing HF compared to LF. Upper body pre- to post-intervention strength gain was similar when MF was compared with LF. Lower body pre- to post-intervention strength gain was greater when HF was compared with MF but not statistically significant. The results of this analysis suggest that only negligible muscular strength increases are made with additional weekly RT sessions. The only findings in this analysis that support a significant relationship between RT frequency and strength gain were MF vs. HF in isolation and multi-joint exercises (ES 0.31; 95% CI 0.05–0.58; *p* = 0.02) and LF vs. HF for upper body (ES 0.48; 95% CI 0.20–0.76) *p* ≤ 0.01). However, readers should interpret these findings cautiously as limited study data were available to assess for a graded response relationship between medium and high frequency.

The differences that exist between Grgic et al. [19] and this review (excluding volume-equated analysis) could be due to confounding factors that may have influenced study outcome reliability. Grgic et al. [19] assessment of the consistency of effects across studies has not been included and is an essential part of the meta-analysis [53]. Unless tests for heterogeneity are performed, it is difficult to determine the findings. The quantity *I*^2^ as in the current review was used to assess heterogeneity among subgroups [53], using only *p* values to decide which scale is more consistent with the data [21] is unsuitable because of the differing and limited number of studies. Likewise, the Benton et al. [26] study included within the Grgic et al. [19] was entered as an RT frequency of 2 vs. 3 days week^− 1^ instead of 3 vs. 4 days week^− 1^. This misrepresentation of study data leads to a detrimental effect on the 2, 3, and ≥ 4 days week^− 1^ RT frequency groups that consequently effects the accuracy of pooled mean ES results. It could be assumed that this accounts for the variances between frequency groups and concerning the two papers strength outcomes.

Rhea et al. [54] conducted a meta-analysis [in part] that sought to quantify the optimum dose response for trained and untrained subjects along the continuum of weekly frequency, volume, and training intensities. Rhea et al. [54] provided evidence that may support the contention that increased weekly training frequency is superior to that of single training sessions per muscle group. The researchers reported that the ES for training frequency was different by training status. Rhea et al. [54] stated that the ES increased for untrained groups as RT frequency increased up to 3 days week^− 1^. However, the trained subject’s ES elicited the most significant strength increases with a weekly training frequency of 2 days week^− 1^ [59]. The RT design for the trained group had increased training volume that may have been too challenging for the untrained subjects. Research by Hoffman et al. [38] and Stowers et al. [60] suggest that trained athletes are possibly closer to their strength potential and that higher training frequencies may evoke more significant strength gains. Moreover, Hoffman et al. [38] and Stowers et al. [60] suggest that smaller muscles produce smaller observed strength gains, and this may require the subjects to have more stimulus or more extended observations before reporting statistically significant differences. Evidence from this analysis did not detect significant variances in strength gain when LF was compared to HF in isolation-only exercises (ES − 0.10; 95% CI − 0.43–0.23; *p* = 0.56). However, limited study data were available to assess for a graded response relationship between lower and high frequency.

### Considerations Towards Weekly Training Frequency and Volume-Equated Studies

Centred upon the available body of evidence from two meta-analyses [19, 53] and other recent studies [61–64], it may be suggested that RT volume is a causal factor to increase muscular strength. Depending upon the subjects training status, additional RT training frequency could attribute to changes in muscular strength for untrained subjects due to increased weekly training volume. Examination of pre- to post-strength gain from volume-equated studies in this analysis was comparable when LF was compared to HF. Therefore, not equating for weekly RT training volume in studies that compare strength gains might be erroneous. Limited extrapolation can be made of the effects on muscle strength due to higher RT frequency or increased weekly RT volume. This is supported by subgroup analysis of volume-equated studies in Grgic et al. [19], and this analysis which did not show a significant effect of RT frequency on pre- to post changes on muscular strength. The ES was similar across lower and higher RT frequency strength outcomes.

A recent study by Colquhoun et al. [64] suggests that additional RT frequency does not lead to further strength improvements when volume and intensity are equated. Male subjects were randomly assigned to either 3 or 6 days week^− 1^ training intervention. Pre-and post-baseline strength measurements after 6 weeks indicated that no significant differences between 3 and 6 days week^−1^. This raises several questions concerning the significance of weekly RT volume rather than RT frequency. A recent analysis that we conducted on weekly set volume [61] suggests that there is a graded dose-response relationship between RT volume and muscular strength gains. We concluded that lower weekly set training produced the smallest pre- to post-training strength differences when compared to medium or higher weekly set training. Further support regarding the importance of weekly training volume on muscle hypertrophy is provided by the meta-analysis of Schoenfeld et al. [62]. From the 15 included studies, a significant effect was reported in muscle size due to increased weekly RT volume. The ES difference between lower and higher volumes equated to a difference of 3.9% strength change. Figueiredo et al. [63] state that volume is the most modifiable variable that has the most evidenced-based response with significant physiological effects on muscle. Future research is required from study designs that equate for RT weekly volume to clarify the effect of RT frequency on strength.

### Strengths and Limitations

This meta-analysis has several strengths that separate it from other previous analyses of training frequency. This analysis attempted to apply more robust criteria to try to control potential confounding variables when comparing the effects of weekly training frequency on strength outcomes. Our intent to create an evidence-based dose-response curve of frequency to strength gain was subverted and resulted in a high- vs. low-frequency comparison for some strength measures. This meta-analysis also considered the possible effects of different sections of the body and the impact it has on strength outcomes on the impact of LF or HF weekly training frequency. The design of this study also differed from others, as it did not cluster outcomes. Instead, data were combined across strength measures to improve external validity. Within our design, we considered and included a multi-level model as a strategy for testing heterogeneity across included studies.

As with previous meta-analytic studies, there were limitations driven by the shortcoming of primary data sources. This present meta-analysis attempted to include relevant and frequently cited research data from high-quality sources, the number of studies was small, and variation existed in the design and control of the included studies. Although every effort was made to include research papers from high-quality sources, the number of suitable studies was limited, and the research designs and control among studies were different. Unfortunately, even when controlling for confounding factors, the low number of studies and sample sizes used in this meta-analysis may exert an effect on estimates of ES. The authors have attempted to ensure that all included studies were appropriate due to the initial screening process. This created difficulty in summarising and interpreting study data.

The validity and utility of this analysis should be evaluated with caution as there are limitations due to the inclusion of combined subject’s characteristics (for example, male-female or trained-untrained). This sampling of mixed gender groups, use of extensive age ranges, use of multiple and different measurements, and the use of various training methods has resulted in a moderately large body of evidence that may be deemed unreliable and not provide answers to strength gain questions for individuals or collectively for groups.

The limitations of previous research by default extend to the present meta-analysis deriving data from that research. Two of the 12 included studies used a randomised control design [11, 52]. The other 10 [17, 22, 44–52] did not use a control group. They used a repeated measures design with baseline measure serving as the control, although baseline measures were not uniformly implemented across those studies. The finding of the present analysis suggests that researchers should be cautious when performing mixed-model meta-analyses (mixed gender subject groups and diverse training groups), as this could limit data analysis and produce spurious conclusions. While studies that combine subjects with differing characteristics can provide useful data, there are at the same time limitations in applicability and relevance. For example, combining males and females in a subject pool or including both trained and untrained in a subject pool (or not fully describing training state) will limit the extent to which the findings may be generalised to either population. When analysing strength gain per exercise, it creates confounding aspects that are difficult to control. For example, different exercises that target the biceps and then measure the strength of a lat pulldown will have a direct effect on the strength measurement. Such designs, common in frequency research, are not as reliable as single model data methods.

It is often stated that the design of RT programs is multifaceted, requiring manipulation of several training variables that interrelate with each other. One of those variables is weekly RT frequency. This is not unique to strength training as the FIT approach to programming, Frequency-intensity-time as variables is commonly taught as axiomatically within physical education and exercise science curricula. However, with further investigation, one finds that any attempt to establish and define an optimal training frequency is undermined by conflicting findings and a lack of clear methodological clarity and consistency from previous study protocols. Those issues with methodological clarity create inadequate data estimations from published studies when performing meta-analyses.

Such equivocality creates conditions under which it is difficult to establish any definitive conclusions. Future investigations and research should be as task-specific as possible and with consideration of training status on test validity [65]. As subjects perform pre- to post-1RM measurements, considerations should be made, as this is a task-specific skill that could incorrectly represent relative increases in strength [66]. Attention should also be made concerning training specific tests and relationships between training frequency and improved 1RM performance. As previously mentioned, there is limited primary data with which to develop an evidence-based consensus regarding the best weekly RT frequency to produce strength gain. More considerable attention needs to be placed upon designing and conducting larger studies using homogenous sample pools (similar biological characteristics and training histories). Increased homogeneity and larger sample sizes would improve primary research but would also strengthen meta-analyses. Replication of studies would also be beneficial and would allow the data and findings to be corroborated.

A better body of research evidence from more studies would also have a profound effect on meta-analyses. Performing meta-analyses on RT variables that were not controlled or inadequately controlled and were conducted in heterogeneous samples is problematic. This is because such weak study designs lead to the exclusion of a significant of extant research publications. This, therefore, leads to variability in methods and results reporting among the best research on the topic remains varied and un-replicated, conclusions from their pooled analysis, while stronger than individual report, remain weak. A significant non-experimental finding of this project was that there was very little experimental evidence of any quality or consistency published related to RT frequency.

## Conclusions

Results from this meta-analysis suggest that no significant effect exists between LF and HF RT on muscular strength gain when the volume is equated. When weekly RT volume was not controlled, results suggest that no significant effect of increased RT frequency on muscular strength gains. Therefore, increased weekly RT volume can be attributed as the principal driver for increased muscular strength. It could be suggested that higher training frequency increases total weekly training volume, which provides a positive adaptive stimulus upon muscular strength. The evidence is progressively mounting that shows increased RT weekly volume is a valuable and beneficial training consideration that can be applied to different populations; healthy, diseased, athletic, or the geriatric. The present analysis identifies several shortcomings in the current scientific literature, as limited evidence can fully establish a graded dose-response relationship between increased frequency and strength. These findings also suggest that due to the absence of quality experimental data, it remains unclear whether RT frequency on its own has effects on muscular strength. Our results point to an evident weakness of the literature and strongly suggest that it is essential that robust studies be conducted to either support or challenge the long-accepted training frequency dogma. The strength of current evidence is still restricted and as such indicates that more investigations and replication studies. This should be from appropriate volume-equated study designs, and comparable subject samples are required to explore the effects of varying weekly RT frequencies adequately. Until better evidence is available, the disagreement between researchers, clinicians, coaches, and trainers will continue, each pointing to evidence that supports their contention. As such, it is crucial that individuals working in the delivery of RT programming use evidence-based recommendations.

## Abbreviations

1RM: 1 repetition maximum
ACSM: American College of Sports Medicine
CI: 95% confidence intervals
days week^− 1^: Days per week
ES: Effect size
HF: High weekly training frequency (≥ 3days per week)
IV: Random-effects inverse variance
LF: Low weekly training frequency (1 day per week)
MF: Medium weekly training frequency (2 days per week)
*n*: Number of subjects
NSCA: National Strength and Conditioning Association
*p*: Probability
PRISMA: Preferred Reporting Items for Systematic Reviews and Meta-Analyses
Q: Cochran’s Q statistic
RAN: Randomised trials
RCTs: Randomised control trials
Reps: Repetitions
RT: Resistance training
SDs: Standard deviations
SEM: Standard error of measurement
SMD: Standardised mean difference
weeks: Weeks trained
